# A Reference Point-Based Evolutionary Algorithm Solves Multi and Many-Objective Optimization Problems: Method and Validation

**DOI:** 10.1155/2023/4387053

**Published:** 2023-01-14

**Authors:** Mohammed Jameel, Mohamed Abouhawwash

**Affiliations:** ^1^Department of Mathematics, Sana'a University, Sana'a 13509, Yemen; ^2^Department of Mathematics, Faculty of Science, Mansoura University, Mansoura 35516, Egypt; ^3^Department of Computational Mathematics Science and Engineering (CMSE), Michigan State University, East Lansing, MI 48824, USA

## Abstract

The integration of a decision maker's preferences in evolutionary multi-objective optimization (EMO) has been a common research scope over the last decade. In the published literature, several preference-based evolutionary approaches have been proposed. The reference point-based non-dominated sorting genetic (R-NSGA-II) algorithm represents one of the well-known preference-based evolutionary approaches. This method mainly aims to find a set of the Pareto-optimal solutions in the region of interest (ROI) rather than obtaining the entire Pareto-optimal set. This approach uses Euclidean distance as a metric to calculate the distance between each candidate solution and the reference point. However, this metric may not produce desired solutions because the final minimal Euclidean distance value is unknown. Thus, determining whether the true Pareto-optimal solution is achieved at the end of optimization run becomes difficult. In this study, R-NSGA-II method is modified using the recently proposed simplified Karush–Kuhn–Tucker proximity measure (S-KKTPM) metric instead of the Euclidean distance metric, where S-KKTPM-based distance measure can predict the convergence behavior of a point from the Pareto-optimal front without prior knowledge of the optimum solution. Experimental results show that the algorithm proposed herein is highly competitive compared with several state-of-the-art preference-based EMO methods. Extensive experiments were conducted with 2 to 10 objectives on various standard problems. Results show the effectiveness of our algorithm in obtaining the preferred solutions in the ROI and its ability to control the size of each preferred region separately at the same time.

## 1. Introduction

Most real-world optimization problems usually contain two or more conflicting objective functions. These objective functions must be optimized simultaneously. This type of problem is known as a multi-objective optimization problem (MOP).

In MOPs with contradictory objectives, a single solution that can be considered the best does not always exist. Instead, a set of solutions represents the best compromises among the different objectives. This set, which belongs to the search space, is known as the Pareto set (or efficient set), whereas its images, which belong to the objective space, are known as Pareto front (PF) [1, 2]. Several evolutionary multi-objective optimization (EMO) algorithms, such as NSGA-II [3], SPEA2 [4], and MOEA/D [5], have been suggested in the past two decades or more. Classical EMO mainly aims to obtain a set of well-converged and well-distributed non-dominated solutions that approach the entire PF [6, 7]. Researchers have devoted their effort to developing algorithms in recent years [8–14].

The proportion of non-dominant solutions rises as the number of objectives increases, which is one of the fundamental problems with all EMO approaches. Due to the insufficient selection pressure caused by a high percentage of non-dominant solutions, the EMO approach cannot advance in finding the optimal spots. Incorporating the decision maker's preferences into the algorithm is a practical way to deal with this issue. A new kind of ranking mechanism [9] can be used to make selection pressure stronger and steer the optimization approach to search in a specified region.

**Figure 1:**
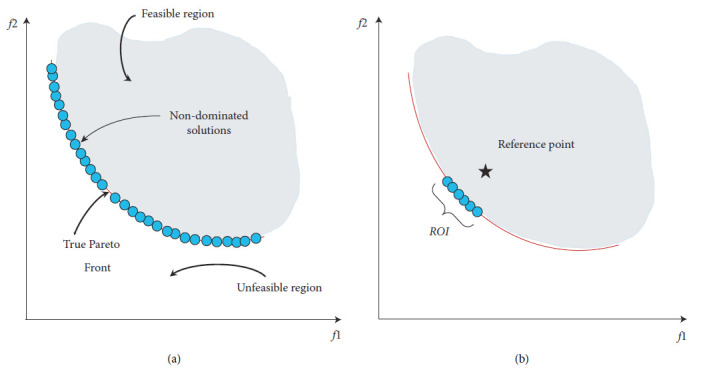
Objective space: (a) evenly distributed across the entire PF; (b) crusting near an RP.

In real life, the DM is always focused on some specified subsets of the obtained solutions. The techniques of preference-based MCDM aim to find a part of the PF, whereas EMO algorithms aim to obtain a well-distributed set of points close to the whole PF. We call the part of the optimal solutions that is near to or lies on the PF a region of interest (ROI) [15]. Solutions within the ROI satisfy the DM's need. However, this scenario does not mean that any efficient solutions outside the ROI are not the optimal solutions to the problem. The preference information given by the DM in the EMO can enable a highly efficient search. Many different approaches to preference information given by DMs exist, such as reference point (RP), preference angle, and reference weights. One of the most utilized approaches in preference-based EMO algorithms is the RP. As mentioned above, EMO tries to find well-distributed multiple efficient solutions across the whole PF, as displayed in Figure 1(a). Also, this figure illustrates the feasible objective region and the unfeasible objective region. On the contrary, preference-based EMO algorithms concentrate on a certain part of the true PF based on a preference point (reference point) determined by DM. The non-dominated points cluster near the RP, as shown in Figure 1(b).

The following is a typical classification of methods based on preferences, depending on how they are expressed by the DM [16–18]: (i) a priori methods, where preferences are expressed before calculating PO solutions, for example, through a utility function [19] or by an RP [20]; (ii) a posteriori methods, in which the DM chooses the solution of her/his preference after a set of efficient solutions has been calculated (for example, [21, 22]); (iii) interactive methods, where the DM guides the search with a utility function, and this function may change during the optimization process because of the new information acquired (for example, [23, 24]); and finally, (iv) methods not based on preferences, where additional information on preferences is not available, and the idea is to find a balance between the objectives [25].

Over the past two decades, researchers have focused their attention on preference-based EMO approaches. These approaches have been actively developed, and they mainly focus on specific parts of the PF. Depending on the preference information supplied by the DM, these algorithms seek to find an ROI that is close to/on the true PF.

Numerous algorithms of preference-based EMO have been introduced. Deb and Sundar [26] suggested the RP-based NSGA-II (R-NSGA-II), which focuses on obtaining a preferred ROI during the evolutionary process. By including the RP's location information in the Pareto dominance, Molina et al. [27] initiated a concept of Pareto dominance termed g-dominance. Ben Said et al. [28] presented a novel variant of the Pareto-dominant relationship, called r-dominance, with which we can obtain good convergence to the PF. Ruiz et al. [29] proposed WASF-GA, another variant of the preference-based MOEA algorithm. Yu et al. [30] suggested a different representative preference-based decomposition MOEA by decomposing the preference information into several scalar optimization problems. Recently, new R-NSGA-II modified methods have been proposed to assist DMs in convergent to Pareto-dominance compliant solutions in a specific area of interest [31–33].

Although many preference-based algorithms use various metrics to select preferred solutions, some of these metrics require prior knowledge of the PF while others require specific parameters [34, 35]. S-KKTPM does not require prior knowledge of the PF or any parameters.

Herein, we introduce a novel preference-based NSGA-II algorithm. The Euclidean distance was utilized in the original R-NSGA-II study as a metric between two trade-off solutions. In our study, we use the simplified Karush–Kuhn–Tucker proximity metric (S-KKTPM) instead of the Euclidean distance metric. S-KKTPM can anticipate the convergence behavior of a point from PF without prior knowledge of the optimum solution [36, 37]. The Karush–Kuhn–Tucker (KKT) conditions occupy a significant role in optimization theory. KKT proximity measure was proposed through these conditions. Incorporating S-KKTPM within the R-NSGA-II provides theoretical convergence properties for the final preferred points. The main contributions of the introduced algorithm are listed below:

- We introduce a new RP-based algorithm called RS-KKTPM, based on the S-KKPM metric, by integrating S-KKPM with NSGA-II to obtain the PO solutions in ROI.
- Obtaining different ranges of ROI in a single run.
- Adding flexibility for several RPs at the same time.
- Obtaining excellent performance when the RP is located in different regions.
- Obtaining a good balance between convergence and diversity aspects around the RP.
- Solving different shapes of PF (e.g., convex, concave, concave, and discontinuous) with a different number of objective functions (up to 10 objectives).
- Making the results competitive compared with those of the other preference mechanisms on many-objective problems.

The layout of this work is as follows.  Section 2 reviews some fundamental definitions. An overview of the works relevant to this paper is mentioned in Section 3. In Section 4, the R-NSGA-II algorithm is combined with the S-KKTPM metric. In the following section, the obtained experiments and the results are discussed and described.  Section 6 summarizes the paper's achievements and presents some upcoming works.  Table 1 displays the nomenclature/abbreviations used in this study.

## 2. Basic Definitions

An MOP contains a set of *n* decision variables, *M* objective functions, *J* constraints of inequality, and *P* constraints of equality. MOP can be defined as follows [28].

(1) $$\begin{array}{c} {Minimize}_{\left(x\right)}f\left(x\right)=\left(\left(f_{1}\right)\right.\left(x\right),\ldots ,f_{M}{\left(x\right)}^{T}\\ subject\;to\;g_{j}\left(x\right)\leq 0,j=1,2,\ldots ,J\text{,}\\ h_{i}\left(x\right)=0,i=1,2,\ldots ,P\text{,} \end{array}$$

where *x*=(*x*_1_,…,*x*_*n*_)^*T*^ is an *n*-dimensional decision variable vector, *f*_*k*_ : *ℝ*^*n*^⟶*R*(*k*=1,2,…, *M*) are the objective functions, and *g*_*j*_ and *h*_*i*_ : *ℝ*^*n*^⟶*R*(*j*=1,2,…, *J*; *i*=1,2,…, *P*) are the constraints of the problem.

In an MOP with contradictory objectives, the search space is only partially ordered, and two solutions may be indifferent to each other. A single decision variable simultaneously optimizing all the objectives is unusual. Consequently, for MOPs, the 〈, 〉, and = operators are extended as follows.

**Definition 1 (Pareto dominance relation).**

Given two solutions **x**, **y** ∈ *ℝ*^*n*^, **x** is said to dominate **y** in the Pareto sense (denoted by *f*(**x**)≺*f*(**y**)) if and only if *f*_*i*_(**x**) ≤ *f*_*i*_(**y**)∀ *i* ∈ {1,…, *M*} and ∃*j* ∈ {1,…, *M*} where *f*_*j*_(**x**) < *f*_*j*_(**y**).

**Definition 2 (non-dominated solution).**

A solution **x** ∈ Ω⊆*ℝ*^*n*^ (Ω is the feasible space) is said to be non-dominated if and only if there does not exist another solution **y** ∈ Ω such that *f*(**x**)≺*f*(**y**).

**Definition 3 (Pareto-optimal (PO)).**

A solution **x** ∈ Ω is said to PO if *f*(**x**) is non-dominated with respect to Ω.

The set of solutions in the search space is called the Pareto solution set (PS). In contrast, the set of all non-dominated vectors in the objective space corresponding to the PS is called the PF [38].

**Definition 4 (PS and PF).**

The PS is defined as follows:

(2) $$\begin{array}{c} PS=\left\{x\in \Omega \;|\;x\;is\;PO\right\}\text{.} \end{array}$$

The corresponding PF is defined as follows:

(3) $$\begin{array}{c} PF=\left\{u=f\left(x\right)\in R^{M}\;|\;x\in PS\right\},\left(R^{M}is\;the\;objective\;space\right). \end{array}$$

**Definition 5 (RP).**

An RP *f*(**x**_*RP*_) is defined in objective space, where *f*(**x**_*RP*_) is provided by the DM.

**Definition 6 (ROI).**

The ROI is the projection of the set of preferred efficient solutions in the objective space, i.e., *ROI*={*f*(**x**) | ‖(*f*(**x**) − *f*(**x**_*C*_)‖ < *δ*, **x** ∈ *PS*}, where *f*(**x**_*C*_) is the closest to the RP *f*(**x**_*RP*_). *δ* denotes the radius of the ROI, which is determined by DM.

## 3. Related Works

In Section 3.1, KKT optimality conditions are briefly reviewed.  Section 3.2 presents R-NSGA-II in detail.

### 3.1. KKT Conditions

KKT conditions play an important role in optimization theory. Through these conditions, it is possible to know if the solution produced by the EMO algorithm is the PO solution or not. For the MOP with inequality constraints, KKT conditions are defined as follows [39]:

(4) $$\begin{array}{c} \overset{M}{\underset{i=1}{\sum }}\beta_{i}\nabla f_{i}\left(x\right)+\overset{J}{\underset{j=1}{\sum }}\gamma_{j}\nabla g_{j}\left(x\right)=0\text{,} \end{array}$$

(5) $$\begin{array}{c} g_{j}\left(x\right)\leq 0,j=1,2,\ldots ,J\text{,} \end{array}$$

(6) $$\begin{array}{c} \gamma_{j}g_{j}\left(x\right)=0,j=1,2,\ldots ,J\text{,} \end{array}$$

(7) $$\begin{array}{c} \gamma_{j}\geq 0,j=1,2,\ldots ,J\text{,} \end{array}$$

(8) $$\begin{array}{c} \beta_{i}\geq 0,i=1,2,\ldots ,M\text{,} \end{array}$$

The parameters *β*_*i*_ and *γ*_*j*_ are called the Lagrange multipliers for the *i*th objective function and *j*th inequality constraint, respectively. Any solution **x** that satisfies each the above conditions is called a KKT point. Equations (4) and (6) are called the equilibrium and complimentary slackness equations, respectively. The conditions stated in equation (5) ensure feasibility for **x** while the conditions stated in equation (7) ensure that the parameters *γ*_*j*_ are non-negative. The conditions stated in equation (8) also ensure that the parameters *β*_*i*_ are non-negative, but at least one of them must be non-zero. In the following section, we briefly discuss R-NSGA-II algorithm.

### 3.2. R-NSGA-II Algorithm

As mentioned in Section 1, classical EMO algorithms mainly aim to develop a finite number of random solutions into a set of non-dominant solutions that converge and distribute across the entire PF over several generations. On the contrary, preference-based algorithms aim to produce non-dominated solutions centered around the desired part (s) of the PF based on the preference information supplied by the DM. This information can be given in several techniques: RPs, aspiration levels, weights, and reference direction [2]. RPs are one of the most used techniques in preference-based EMO algorithms. Usually, an RP is said to be achievable if it lies in the feasible objective space; otherwise, it is said to be unachievable.

In 2006, Deb and Sundar [26] put forward R-NSGA-II method, which presents the DM's preferences as one or more RPs. The method is based on the benchmark manner, which is based on preference information [40]. It is a modification of the widely used EMO approach NSGA-II, in which an Euclidean distance metric is applied instead of the crowding distance metric from the RP that indicates DM's preference. The primary notion behind R-NSGA-II is to give preference to parents who have short Euclidean distances to the RP. The following is the description of the R-NSGA-II procedure: *P*_*t*_ (of size *N*) is a randomly generated parent population. A new offspring population *Q*_*t*_ (of size *N*) is generated using the number of operations (binary tournament selection, recombination, and mutation). Thereafter, the populations *P*_*t*_ and *Q*_*t*_ are combined, and the resulting population *R*_*t*_ = *P*_*t*_ + *Q*_*t*_ (size 2*N*) is classified according to dominance in fronts. The new population *P*_*t*+1_ is built starting with the fronts with the lowest rank until reaching a front *F*_*i*_, which cannot be accepted without making the size of population to exceed *N*. Next, the preference operator is applied to the front *F*_*i*_ to maintain the size of the new population. The final front *F*_*i*_, which cannot be fully accepted, is then considered, and the remaining slots are filled according to an environmental selection approach. The Euclidean distance for each RP is calculated with respect to each solution of the front *F*_*i*_. For each RP, the solution closest to the said point takes the preferred distance value of 1. The solutions that are closest to all of the RPs are given the shortest preferred distance. The preferred distance value of 2 is then applied to the solutions with the next smallest distance to each RP, and the process is repeated for the remaining *F*_*i*_ solutions. In the generation of the new population of descendants, the preferred solutions in the selection by the tournament are those with a lower value of preferred distance.

The idea of *ϵ*-*based* *selection* *strategy* is utilized to maintain diversity in the solutions close to each RP. First, a solution of the front *F*_*i*_ is randomly chosen. Next, the Euclidean distance in the objective space of all the solutions is computed with respect to the chosen solution. After that, the points that have a sum of the normalized difference in the objective search space values less than or equal to *ϵ* from the selected point are given an artificial large distance to remove them from the competition; in this method, only a solution within *ϵ*-neighborhood is relevant. The process continues by randomly choosing another solution different from the previous one, to which the concept of *ϵ*−based selection strategy described above is applied again.

#### 3.2.1. Advantages and Disadvantages

Compared with classical RP-based algorithms, R-NSGA-II works well for high-dimensional MOPs; it is suitable for any frontier shape, several objectives, and variables. It also shows some advantages: the classical methods depend on the reference direction (weight vector); however, R-NSGA-II is independent of the weight vector. Moreover, the classical methods in most cases can only find efficient solutions for different RPs by applying the algorithm to each RP for several times, whereas R-NSGA-II can produce a set of efficient points for different RPs in a single simulation. RPs can exist anywhere in the objective space (achievable or unachievable). However, it requires a parameter *ϵ* to maintain a diversity of selected solutions near the RPs.

As mentioned above, the crowding distance metric of NSGA-II has been replaced using the Euclidean distance metric in R-NSGA-II to obtain the solutions closest to the RPs assigned by DMs. However, the final minimal Euclidean distance value is unbeknown. Thus, ascertaining whether the efficient solution is accomplished at the end of an optimization run is difficult. In other words, the Euclidean distance metric does not have any information about the proximity of a solution to the PF. Additionally, in the case of achievable RPs, the Euclidean distance metric may not be monotonically reduced to its minimum value. One major disadvantage of this method is that the DM cannot control the size of each preferred region separately. Furthermore, the DM cannot smoothly control the obtained PO solutions within each desired region. Below, we introduce a new approach that is based on integrating the S-KKTPM metric with the R-NSGA-II algorithm.

## 4. The Introduced R-NSGA-II with S-KKTPM

In Section 4.1, the development of the KKT-proximity measure is introduced.  Section 4.2 presents the proposed RS-KKTPM in detail.

### 4.1. S-KKTPM

KKT conditions are necessary to know whether the solution obtained by the EMO algorithm is a KKT point. Hence, they play an important role in optimization theory [39, 41]. During the last decade, a KKT-proximity measure has been developed utilizing KKT optimality theory. In 2013, a KKT-based proximity metric (KKTPM) was suggested by Dutta et al. [42] to calculate a KKTPM value for any iteration (or solution) **x**^*k*^ for a single-objective optimization problem. Deb and Abouhawwash et al. [37, 43] extended the above KKTPM for MOPs. Their expansion, which is based on the incorporation of the KKTPM metric via scalarization approaches, aims to relate the convergence property of a solution from a specific optimal solution. Other information on KKTPM for MOPs can be found in [37, 43].

**Figure 2:**
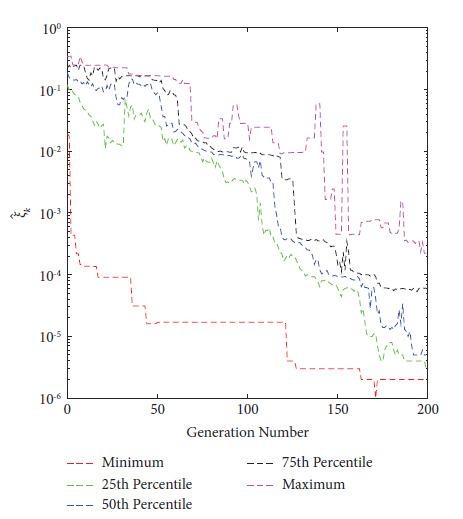
$\overset{\wedge }{\xi_{k}}$ values versus generations for unconstrained ZDT1 problem.

**Figure 3:**
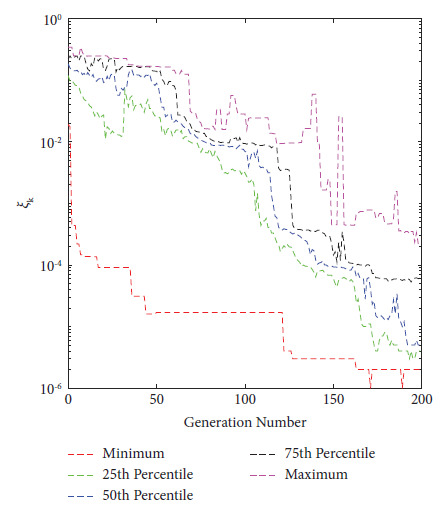
*ξ*_*k*_ values versus generations for unconstrained ZDT1 problem.

**Figure 4:**
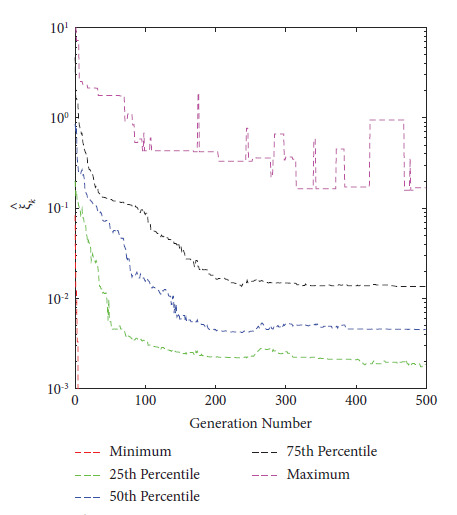
$\overset{\wedge }{\xi_{k}}$ values versus generations for constrained SRN problem.

**Figure 5:**
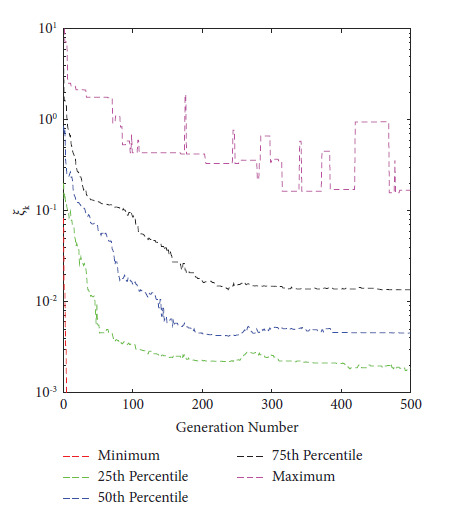
*ξ*_*k*_ values versus generations for constrained SRN problem.

In 2021, Eichfelder and Warnow [44] proposed a new KKTPM metric for MOPs without using any scalarization approach. The authors defined the following methodology for calculating the KKTPM value for any solution **x**^*k*^ ∈ *ℝ*^*n*^, for the MOP mentioned in equation (1):

(9) $$\begin{array}{c} {minimize}_{\left(\xi_{k},\beta ,\gamma \right)}\xi_{k}\text{,}\\ subject\;to{\left\|\overset{M}{\underset{i=1}{\sum }}\beta_{i}\nabla f_{i}\left(x^{k}\right)+\overset{J}{\underset{j=1}{\sum }}\gamma_{j}\nabla g_{j}\left(x^{k}\right)\right\|}^{2}\leq \xi_{k}\text{,}\\ \overset{J}{\underset{j=1}{\sum }}\gamma_{j}g_{j}\left(x^{k}\right)\geq -\xi_{k}\text{,}\\ g_{j}\left(x^{k}\right)\leq \xi_{k},\forall j,\\ \overset{M}{\underset{j=1}{\sum }}\beta_{i}=1\text{,}\\ \beta_{i}\geq 0,\forall i\;and\;\gamma_{j}\geq 0,\forall j\text{,} \end{array}$$

where *J* and *M*, respectively, are the numbers of constraint and objective functions. The value *ξ*_*k*_ obtained after the optimization is the KKTPM at the point **x**^*k*^. First-order derivatives of constraint and objective functions are necessary to solve this problem. KKTPM metric can be utilized to single, multi, and many-objective optimization problems. The above problem has (*M*+*J*+1) variables, (*M*+2*J*+2) inequality constraints, and one equality constraint. To reduce the number of constraints in the optimization problem mentioned above, we propose to redefine it as follows:

(10) $$\begin{array}{c} {minimize}_{\left(\overset{\wedge }{\xi_{k}},\overset{\wedge }{\beta },\overset{\wedge }{\gamma_{j}}\right)}\overset{\wedge }{\xi_{k}}\text{,}\\ subject\;to{\left\|\overset{M}{\underset{i=1}{\sum }}\overset{\wedge }{\beta_{i}}\nabla f_{i}\left(x^{k}\right)+\overset{J}{\underset{j=1}{\sum }}\overset{\wedge }{\gamma_{j}}\nabla g_{j}\left(x^{k}\right)\right\|}^{2}\leq \overset{\wedge }{\xi_{k}}\text{,}\\ \overset{J}{\underset{j=1}{\sum }}\overset{\wedge }{\gamma_{j}}g_{j}\left(x^{k}\right)\geq -\overset{\wedge }{\xi_{k}}\text{,}\\ \overset{M}{\underset{j=1}{\sum }}\overset{\wedge }{\beta_{i}}=1\text{,}\\ \overset{\wedge }{\beta_{i}}\geq 0,\forall i\;and\;\overset{\wedge }{\gamma_{j}}\geq 0,\forall j\text{,} \end{array}$$

where the variable vector of the above optimization problem is $\left(\overset{\wedge }{\xi_{k}},\overset{\wedge }{\beta },\overset{\wedge }{\gamma }\right)$. The value of $\overset{\wedge }{\xi_{k}}$, which solves the above problem, is referred to as the simplified KKTPM (S-KKTPM). The primary goal of reducing the number of constraints is to save the computational cost of an optimization problem. The above problem has (*M*+*J*+1) variables, (*M*+*J*+2) inequality constraints, and one equality constraint. The number *J* of inequality constraints has been reduced compared to the optimization problem mentioned in equation (9) without affecting the optimization process. To ensure that values of both $\overset{\wedge }{\xi_{k}}$ and *ξ*_*k*_ obtained after the optimization are identical at point **x**^*k*^, first we consider the ZDT1 unconstrained problem with thirty variables [45]. We ran NSGA-II for 200 generations in this problem, with a population size of 40. Figures 2 and 3 illustrate the $\overset{\wedge }{\xi_{k}}$ and *ξ*_*k*_ values versus generation numbers for efficient solutions to the unconstrained ZDT1 problem. The minimum, 25^th^ percentile, 50^th^ percentile, 75^th^ percentile, and maximum $\overset{\wedge }{\xi_{k}}$ and *ξ*_*k*_ values are also plotted for all PO solutions at each generation. Both figures show a constant reduction as the generation number increases. With a correlation coefficient of 0.9996, both figures show that the values and patterns of $\overset{\wedge }{\xi_{k}}$ and *ξ*_*k*_ are identical. Second, we consider the SRN unconstrained problem with two variables and two constraints [46]. In this problem, we also ran NSGA-II until generation 500, with a population size of 200. $\overset{\wedge }{\xi_{k}}$ and *ξ*_*k*_ values versus generations for obtained solutions are displayed in Figures 4 and 5. Both figures also show that the values and patterns of $\overset{\wedge }{\xi_{k}}$ and *ξ*_*k*_ are congruent, with a correlation coefficient of 0.9999.

**Figure 6:**
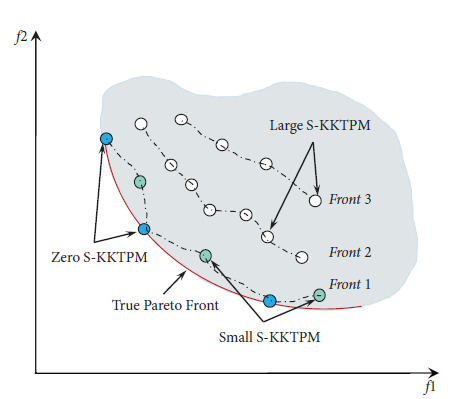
S-KKTPM values for a set of non-dominated points.

An advantage of S-KKTPM metric given in equation (10) is that it predicts the convergence behavior of a point from the PF without prior knowledge of the PO solution. Now, we describe several features of the S-KKTPM [37, 43, 44]:

- It can be utilized as a termination condition for the algorithm of optimization.
- It is applicable in high-dimensional MOPs; S-KKTPM is suitable for any frontier shape, large number of objectives, and variables.
- It provides a monotonous characteristic of the S-KKTPM surface over the objective space. S-KKTPM value decreases monotonously almost to zero as the iterate approaches the efficient solution.  Figure 6 displays the S-KKTPM values for a set of efficient solutions located at different positions in objective space; for example, S-KKTPM value is zero in the true PO solutions (marked by blue circles), which lie on the PF. For efficient solutions, which are close to the PF (marked by green circles), S-KKTPM value is small. For far-away solutions from the PF (marked by white circles), S-KKTPM value is large.
- Calculating S-KKTPM value does not require any parameters, such as weight vector and ideal point, unlike when calculating values in other versions of KKT proximity measure.

In this study, we use S-KKTPM optimization problem to calculate $\overset{\wedge }{\xi_{k}}$ value at iterate **x**^*k*^. We used MATLAB fmincon() algorithm optimization to solve S-KKTPM optimization problem (see Algorithm 1).

### 4.2. The Proposed RS-KKTPM

To make R-NSGA-II solutions preferred and acceptable to DMs and to easily control the size of each region, S-KKTPM metric is integrated with the-NSGA-II algorithm.

In this study, we refer to the RP-based S-KKTPM as RS-KKTPM. The introduced algorithm allows DMs to apply any number of RPs. RS-KKTPM also allows the DMs to control the size of the preferred parts separately. In the introduced algorithm, we replace the Euclidean distance metric, utilized in R-NSGA-II, with S-KKTPM metric. Solutions with small S-KKTPM values are chosen in the introduced method. The preference operator is utilized in this algorithm to select a subset of solutions from the final front that cannot be accommodated totally to maintain the size of population in the novel population. Instead of using the preference distance as in R-NSGA-II, this preference operator uses the preference S-KKTPM metric.

We now characterize an iteration of the introduced R-NSGA-II with S-KKTPM process in which the DM provides one or more RPs in the following section (see Algorithm 2). Both parents and children are merged as usual, and the non-dominated sorting strategy is employed to classify the merged population into non-domination levels (so-called fronts).

The following are the primary ideas underlying selecting the preferred set of solutions within the preferred range:

- Solutions closest to RP are always prioritized.
- Preferred-region sizing strategy is used to control the preferred range near RP.
- *ϵ*-based selection strategy is utilized to keep the spread of solutions within the range assigned by the DMs.

**Figure 7:**
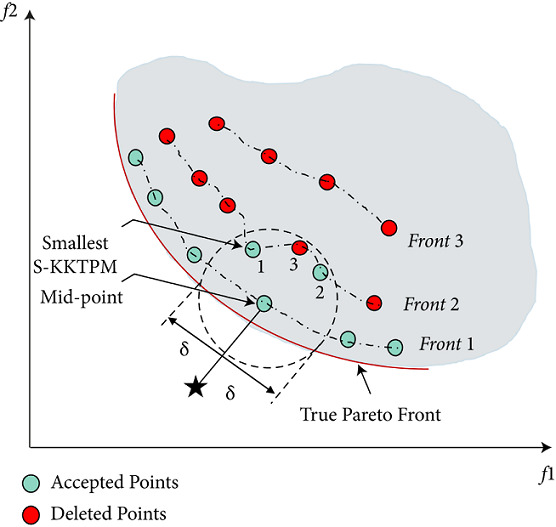
S-KKTPM-based metric in R-NSGA-II procedure. Numbers indicate the assigned ranks of points.

The following changes are made to the original NSGA-II niching approach to integrate the three notions mentioned above:

 
*Step 1*. Generating a desired region for each RP. The Euclidean distance between all members from the merged population and an RP is computed to specify the desired region. Then, the member that has the least Euclidean distance to RP is identified. The specified member (or point) is called mid-point as illustrated in Figure 7.
 
*Step 2*. Determining the size of the desired region for each RP. Here, we introduce a new strategy to determine the size of each desired area as follows. The solution within *δ* distance of the mid-point is chosen to be in the desired area. Parameter *δ* is given by the DM, which determines the size of the ROI, as illustrated in Figure 7. This figure also shows how to choose a population of size eight from the merged population containing 17 members. All solutions in the first front are selected, as shown in Figure 7. Then, we need only two solutions from the second front. The remaining two solutions are chosen (from the second front) as follows. The S-KKTPM value is calculated for each solution (**x**) within the ROI. Then, the minimum of the appointed ranks is appointed as the S-KKTPM value to a solution (**x**). If the solution (**x**) is not within the preferred region, we set a high value for S-KKTPM (see Algorithm 3). In this manner, the smallest S-KKTPM value of one is given to the points that are closest to the PF. The next-to-smallest S-KKTPM value of two is given to the solutions with the next-to-smallest S-KKTPM value to the true PF, and so on. Finally, the solutions with the smallest S-KKTPM are preferred to survive and transition to the new population.
 
*Step 3*. Good distribution of the obtained solutions. The *ϵ*-clearing selection strategy, employed in the original R-NSGA-II, is used in RS-KKTPM to control the diversity of chosen solutions near the RPs. A solution is selected randomly from the set of non-dominated solutions to implement this strategy. Then, any solution with a sum of normalized differences in objective values less than *ϵ* is selected and then given a high-preference distance value to discourage it from remaining in the next generations of the evolution process. The way is then repeated with a new solution picked from the set of efficient points (excluding the one previously selected). The value of *ϵ* is selected according to the application and can be different for each objective. Thus, it is formed as a parameter provided by the DMs.

**Figure 8:**
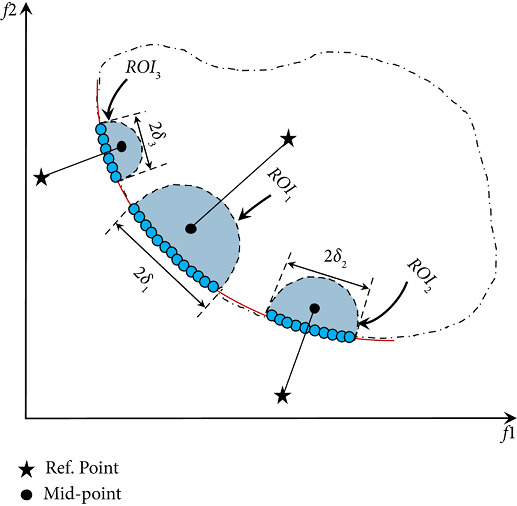
ROIs for DM with different ranges.

Figure 8 depicts how to determine the size of the ROI for each RP using the *mid-point* strategy. As discussed in step 1 above, the *mid-point* is a member of the population that is closest to the RP. As shown in Figure 8, RP can exist anywhere in the objective region (feasible or unfeasible), whereas the *mid-point* can exist anywhere in the feasible objective domain only. The purpose of the proposal of *mid-point* strategy can be summarized as follows: (1) getting PO solutions that are close to the given RP; (2) determining the size of the ROI by calculating the Euclidean distance between each solution and the *mid-point* (each distance value is normalized using zero as the lower bound and one as the upper bound to stay within the interval [0, 1]; the solutions that lie within *δ* value are candidates to be within the ROI); and (3) obtaining a good convergence of solutions towards the ROI. As discussed in step 2 above, the S-KKTPM metric acts as a differentiator in selecting a solution that should remain in the next generations of the optimization process. The solution with the smallest S-KKTPM value is preferentially kept for the next generations because it is the closest to the true PF. This way, the RS-KKTPM can obtain good convergence of solutions towards the ROI. The introduced algorithm can well distribute solutions along the preferred part. RS-KKTPM works well with different RPs (feasible or infeasible) in the objective space, as displayed in Figure 8. In real-world applications, objectives should be normalized when they do not have the same units. Otherwise, *δ* is not a meaningful parameter.

One of the essential advantages of the introduced method is its ability to control the size of the preferred areas separately by a single simulation run (see Figure 8). This is done using the preferred-region sizing strategy discussed above, based on the S-KKTPM metric. This metric is used as a preference operator to select a subset of solutions close to the PF in order to move to the next population. As the iteration approaches the PF, S-KKTPM value decreases monotonically almost to the final minimum value (zero). This means that the S-KKTPM metric can know the proximity of a point in the search space to the PF. Through this strategy, the introduced algorithm can steer the solutions during the optimization process towards the preferred regions in proportion to the size of each area. In other words, the large ROI gets more PO solutions compared to the smaller preferred region.

On the other hand, the original R-NSGA-II algorithm cannot control the size of the preferred regions separately through a single run. The reason is the preferred-region sizing strategy used in this algorithm, which is based on the Euclidean distance metric. This metric is utilized as a preference operator in the R-NSGA-II algorithm. However, the Euclidean distance metric does not have the unique properties that the S-KKTPM metric does. For example, the final minimal Euclidean distance value is unknown. In other words, the Euclidean distance metric does not have any information about the proximity of a point to the PF. So, the R-NSGA-II algorithm cannot obtain different ranges of ROI in a single run.

## 5. Experimental Results and Discussion

This section uses a set of benchmark problems and engineering design problems to test our introduced methodology. Specifically, we adopted five two-objective unconstrained problems taken from the ZDT test suite [45], four bi-objective constrained problems (BNH, SRN, OSY, and TNK) taken from [46], and seven test problems having from three to ten objective functions taken from the DTLZ test suite [47]. In addition, we adopted two engineering design problems, the welded beam design problem with two objective functions (taken from [48]) and the car side impact design problem with three objective functions (taken from [49]). Then, we compare the performance of the RS-KKTPM approach with six EMO preference approaches, including R-NSGA-II, g-NSGA-II [27], r-NSGAII [28], R-NSGA-III [50], WV-MOEA-P [51], and MOEA/D-PRE.

The parameters of the suggested method are set as follows:

- Reproduction operators: as suggested in original study [26], simulated binary crossover (SBX) probability and SBX index are set to 0.9 and 10, respectively, and the polynomial mutation probability and mutation index are set to 1/*n* and 20, respectively.
- Population size, maximum number of generations, RPs, and size of ROI (*δ*): different parameters for a set of different test instances are displayed in Tables 2 and 3.

For constraint handling in constraint test problems and engineering design problems, we handled it by adding a penalty proportional to the constraint violation to the objective function value as suggested in the original NSGA-II algorithm. In minimization problems, this is a popular approach to deal with constraints in evolutionary algorithms.

The proposed RS-KKTPM algorithm is implemented in the MATLAB R2019a platform. The source codes for the comparison methods are provided by PlatEMO [51] or downloaded from the authors' home page. The suggested and compared methods are simulated on a personal computer with an Intel(R)Core(TM)i7-7500 2.9 GHz Quad-Core Processor and 8 GB RAM.

### 5.1. Experiments on Two-Objective Unconstrained ZDT Problems

**Figure 9:**
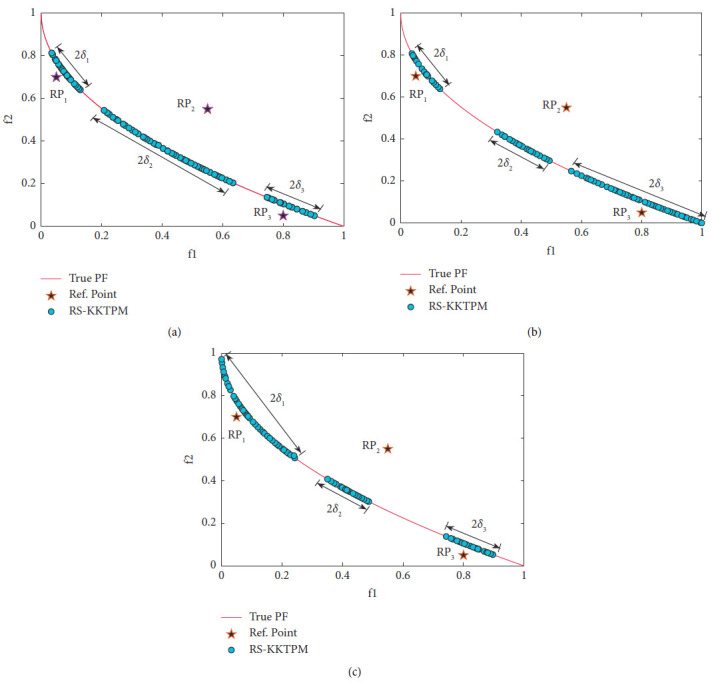
Effect of *δ* in obtaining different sizes of desired regions on ZDT1. (a) *δ*_1_=0.05; *δ*_2_=0.15; and *δ*_3_=0.05. (b) *δ*_1_=0.05; *δ*_2_=0.05; and *δ*_3_=0.15. (c) *δ*_1_=0.15; *δ*_2_=0.05; and *δ*_3_=0.05.

Now, we apply our proposed approach to ZDT1 unconstrained problem (it has a convex PF) with thirty variables.  Figure 9 illustrates the influence of different values of *δ* on the distribution of solutions obtained by RS-KKTPM after 200 generations (i.e., 16000 evaluations, given that RS-KKTPM evaluates 80 offsprings per generation). Three RPs are chosen: *RP*_1_=(0.8,0.05), *RP*2=(0.55,0.55), and *RP*_3_=(0.05,0.7). These RPs are shown in the filled stars. *RP*_1_ and *RP*_3_ lie in infeasible search space while *RP*_2_ lies in feasible search space. The different values of *δ* corresponding to the RPs are detailed in Figures 9(a)–9(c).

**Figure 10:**
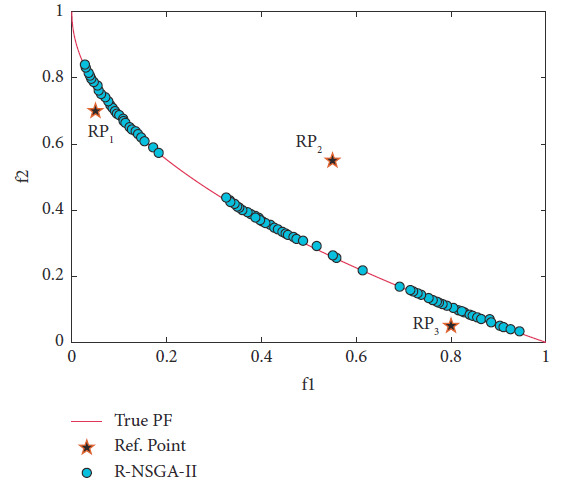
Efficient solutions obtained by R-NSGA-II for three RPs on ZDT1.

Through different values of *δ*, the proposed algorithm can steer the solutions towards the preferred regions in proportion to the size of each region. Parameter *ϵ* is still required to ensure that the obtained solutions are well distributed within preferred region. In this problem, the parameter *ϵ* = 0.005 is chosen. The solutions obtained are clustered near the RPs, as shown in Figures 9(a)–9(c). The distribution of the obtained PO set depends on the range of each desired region. In particular, the range of solutions obtained is equally vast when the value of *δ* is large. One of the advantages of RS-KKTPM is that it allows us to adjust the ranges for the desired region in a single run. Thus, if the DM wants to get a set of solutions (near each preferred region) whose number varies depending on the size of each preferred region separately, different values of *δ* can be chosen. In other words, the DM can control the spread of the generated ROIs by changing the value of parameter *δ*. If *δ* = 0.5, the RS-KKTPM provides an approximation of the entire PF. On the contrary, Figure 10 shows the PO set produced utilizing R-NSGA-II for the same three RPs on ZDT1 problem. R-NSGA-II is also performed with *ϵ* = 0.005 and a population of size 80. It is run until 200 generations. Figure 10 shows that the DM (by RNSGA-II) cannot obtain different regions of desired regions in a single run. Also, R-NSGA-II cannot steer the solutions toward the preferred regions in proportion to the size of each region.

**Figure 11:**
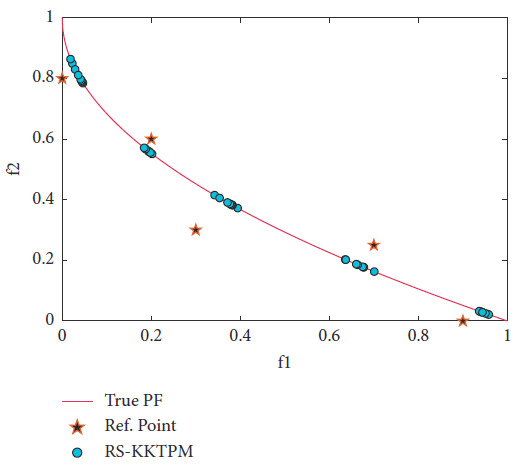
Efficient solutions obtained by RS-KKTPM on ZDT1 with five RPs.

Henceforth, the parameter *ϵ* = 0.001 is used in all problems. First, we consider ZDT1 test problem with five RPs, of which three are infeasible and two are feasible, as shown in Figure 11. Each RP and corresponding size of ROI are shown in Table 2. RS-KKTPM is utilized for this problem, where the population members and the maximum number of generations are 40 and 200, respectively. The parameter is set to 0.05 for each ROI. Figure 11 also demonstrates how easy the proposed algorithm can be modified to address multiple RPs. As a result, it discovers various ROIs. Well-convergent non-dominated solutions are obtained on PF near all the five RPs.

**Figure 12:**
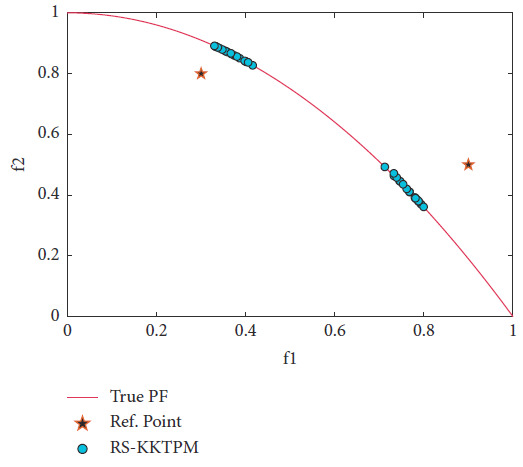
Efficient solutions obtained by RS-KKTPM on ZDT2 with two RPs.

ZDT2 is the next problem which has a non-convex PF. Two RPs are chosen, of which one is feasible and the other is infeasible, as presented in Table 2. The range of each region corresponding to an RP is also presented in Table 2. The population members and maximum number of generations, respectively, are 40 and 200.  Figure 12 displays the convergence and distribution of the solutions near the two chosen RPs. As shown in the figure, RS-KKTPM algorithm can easily deal with feasible and infeasible RPs. The proposed algorithm proves its ability to converge and distribute the solutions obtained within the desired ranges provided by the DMs, as illustrated in Figure 12. RS-KKTPM also showed good distribution on this problem when RP is in the infeasible region.

**Figure 13:**
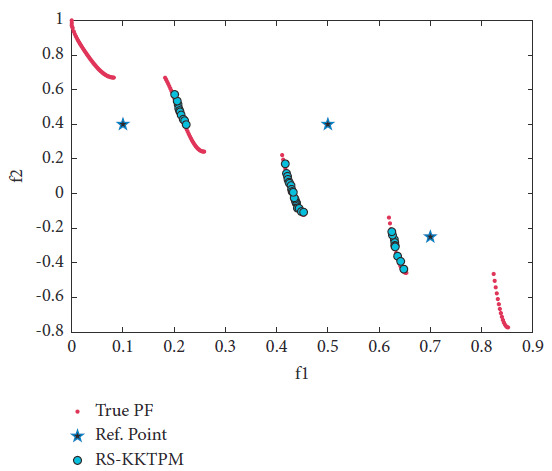
Efficient solutions obtained by the RS-KKTPM on ZDT3 with three RPs.

**Figure 14:**
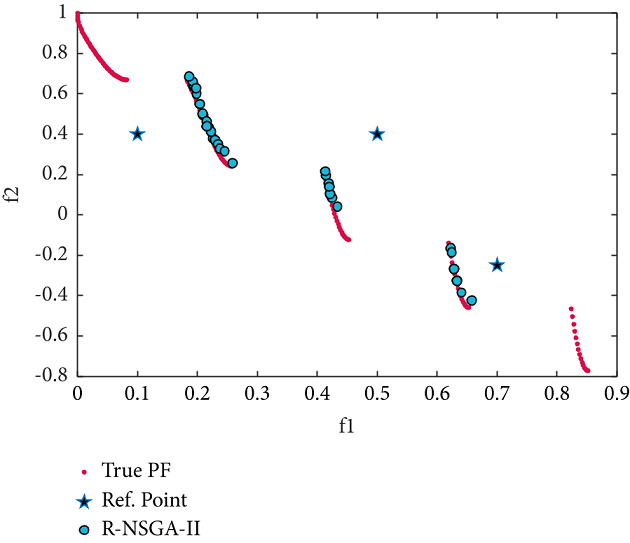
Efficient solutions obtained by R-NSGA-II on ZDT3 with three RPs.

The test problem ZDT3, with 30 variables, has a disconnected set of PFs. Three RPs are selected (see Table 2), of which one is infeasible and two are feasible. The desired solutions produced by RS-KKTPM and R-NSGA-II are illustrated in Figures 13 and 14. The population members were 40, and the maximum number of generations was 200. These two figures demonstrate that our approach is able to steer solutions towards the PF in proportion to the size of each ROI, while the R-NSGA-II cannot. As illustrated in Figure 13, our approach does not get stuck in any locally PO part, and all generated solutions are non-nominated and global PO solutions.

**Figure 15:**
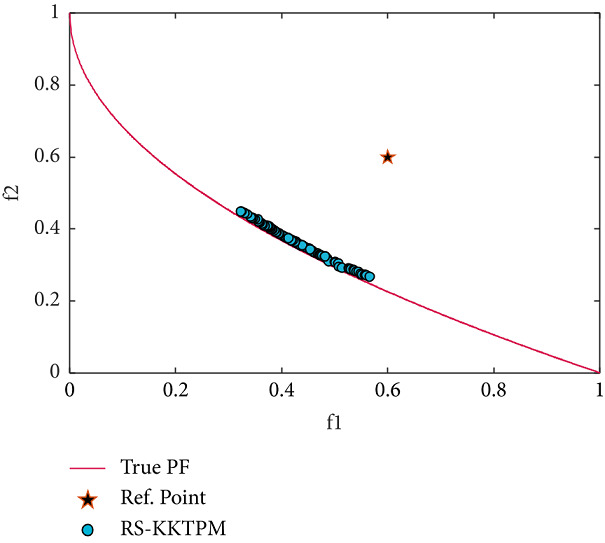
Efficient solutions obtained by RS-KKTPM on ZDT4 with one RP.

**Figure 16:**
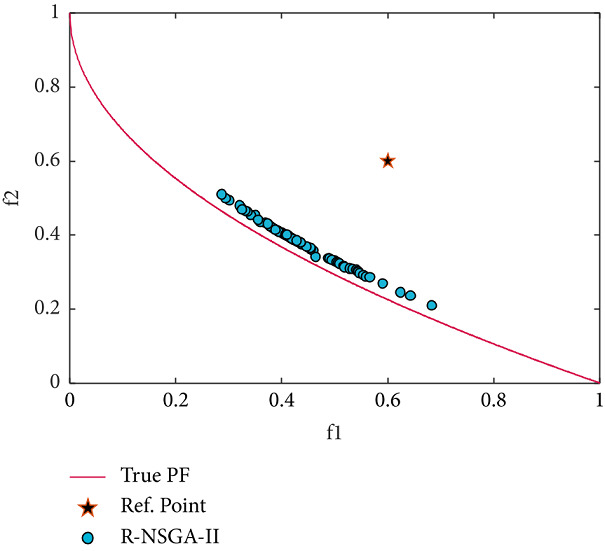
Efficient solutions obtained by R-NSGA-II on ZDT4 with one RP.

Next, the test problem of ZDT4 with 10 variables is solved utilizing RS-KKTPM and R-NSGA-II. This problem has many local PFs. One RP is used with a range of ROI of 0.15, as displayed in Table 2. The RP is (0.6, 0.6), and the generations are 500. The plot of the desired solutions produced by RS-KKTPM and R-NSGA-II is represented in Figures 15 and 16, respectively. As illustrated by the two figures, the performance of RS-KKTPM is much better than that of R-NSGA-II in terms of the distribution and convergence of solutions from the PF. As shown in Figure 15, the selected RP is somewhat far from the PF, which indicates the ability of the introduced approach to work well in the case of distant RPs. Thus, as shown in Figure 15, even though the problem has more than 100 local fronts, the introduced algorithm can converge well to the true PF.

**Figure 17:**
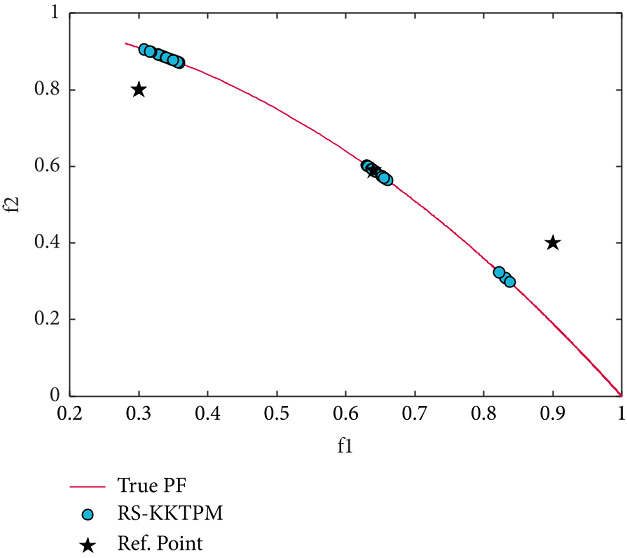
Efficient solutions obtained by RS-KKTPM on ZDT6 with three RPs.

**Figure 18:**
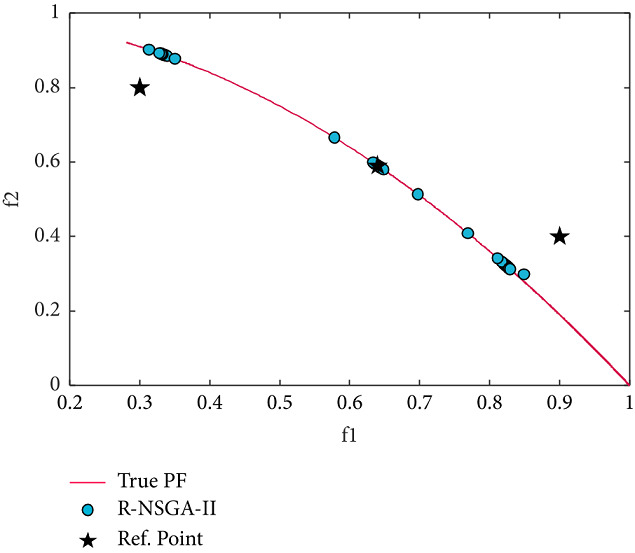
Efficient solutions obtained by R-NSGA-II on ZDT6 with three RPs.

Finally, we apply our proposed method to a ZDT6 problem that has a non-convex PF. Figures 17 and 18 display the obtained PO solutions by RS-KKTPM and R-NSGA-II, respectively. Both techniques used the same RPs, the same number of population members, and the same number of generations (see Table 2). Three RPs are chosen, of which the first lies in feasible search space, the second lies close to/on PF, and the third lies in infeasible search space. For RS-KKTPM, the sizes of ROIs corresponding to RPs (0.9, 0.4), (0.3, 0.8), and (0.64, 0.59) are 0.03, 0.05, and 0.10. For R-NSGA-II, the size of ROIs for all RPs is 0.10. Note that R-NSGA-II cannot adjust the size of each ROI separately, as in RS-KKTPM. As it is clear from Figure 17, the introduced algorithm can seek solutions towards the ROI in proportion to the size of each area separately, whereas R-NSGA-II cannot. The ROI corresponding to RP (0.64, 0.59), with *δ*=0.1, contains a large number of points compared to the ROI corresponding to RP (0.3, 0.8) with *δ*=0.05, as shown in Figure 17. Also, the ROI corresponding to RP (0.9, 0.4), with *δ*=0.03, contains a few number of solutions compared to the ROIs corresponding to RPs (0.3, 0.8) and (0.64, 0.59). This means that if the DM wants to get PO solutions of different sizes for all regions, the introduced algorithm can do that. In contrast, R-NSGA-II cannot control the number of solutions for each desired area. This is because parameter *ϵ* takes only one value for all preferred regions corresponding to the given RPs. In other words, when multiple RPs exist, R-NSGA-II cannot give different values for *ϵ* in a single run. This means that if the DM wants to get PO solutions of different sizes for all regions, the R-NSGA-II algorithm cannot do that.

### 5.2. Experiments on Two-Objective Constraint Problems

**Figure 19:**
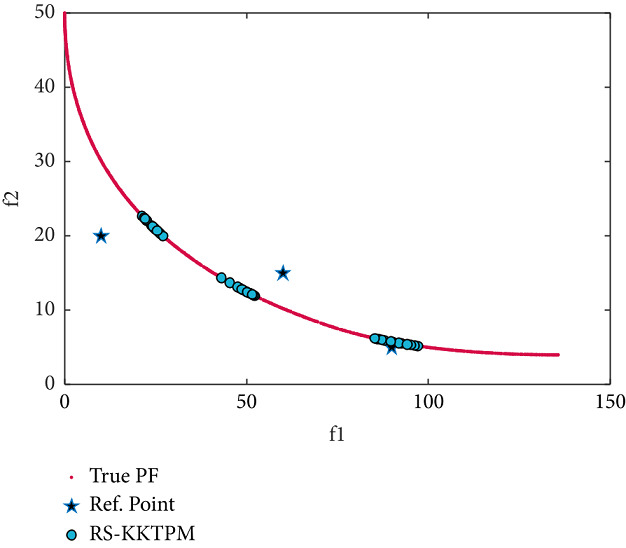
Efficient solutions obtained by RS-KKTPM on BNH with three RPs.

**Figure 20:**
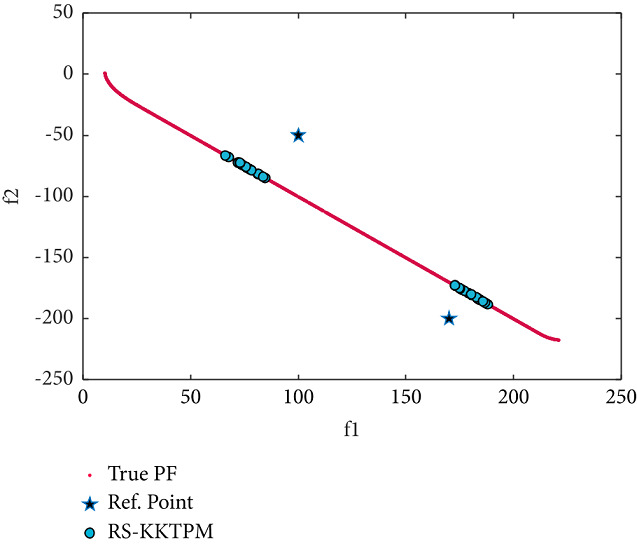
Efficient solutions obtained by RS-KKTPM on SRN with two RPs.

**Figure 21:**
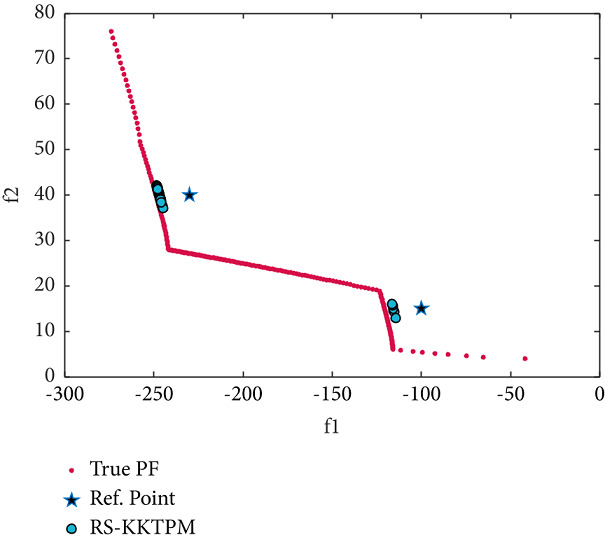
Efficient solutions obtained by RS-KKTPM on OSY with two RPs.

**Figure 22:**
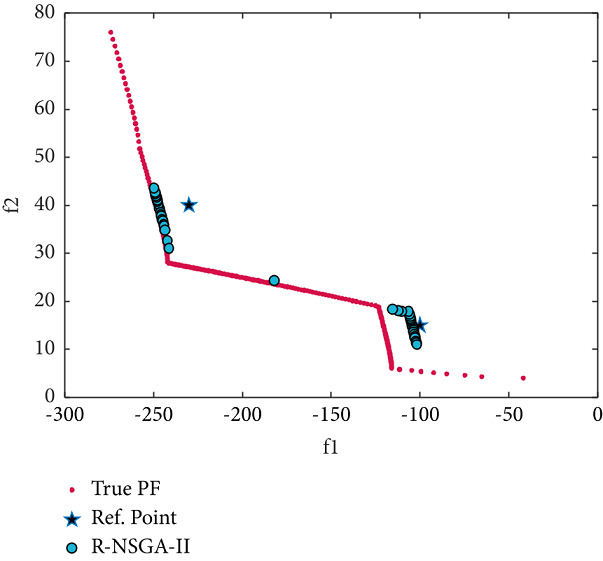
Efficient solutions obtained by R-NSGA-II on OSY with two RPs.

We now consider two-objective constraint problems: BNH, SRN, OSY, and TNK [46]. RPs and some essential parameters used to solve these problems are shown in Table 2. BNH, TNK, and SRN have only two constraints and two variables. First, the efficient solutions obtained by the RS-KKTPM on BNH with three RPs are illustrated in Figure 19. It is clear from this figure that our approach can find the desired regions near the RPs. Second, the solutions obtained on SRN with two RPs are displayed in Figure 20. The RS-KKTPM algorithm works well when the RP is in the feasible or infeasible domain, as displayed in Figure 20. Next, we consider the OSY test problem, which has six constraints and six variables. Figures 21 and 22 show the obtained solutions by RS-KKTPM and R-NSGA-II on OSY, respectively. Two RPs are chosen with a range of ROIs and a population = 40 (see Table 2). Although RS-KKTPM cannot converge to the true PF, it converges slightly better than R-NSGA-II, as shown in Figures 21 and 22.

**Figure 23:**
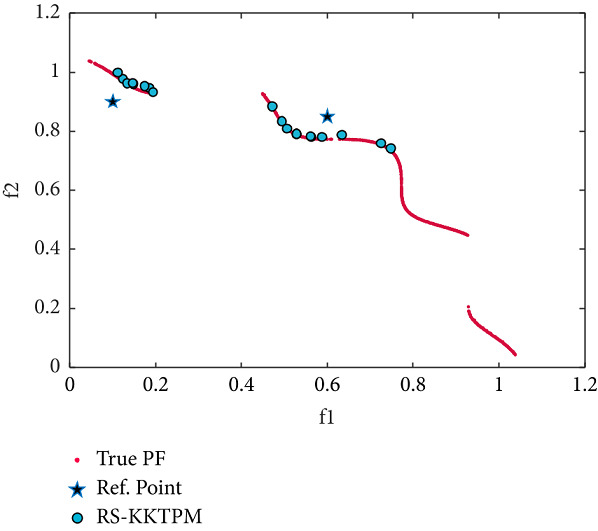
Efficient solutions obtained by RS-KKTPM on TNK with two RPs.

**Figure 24:**
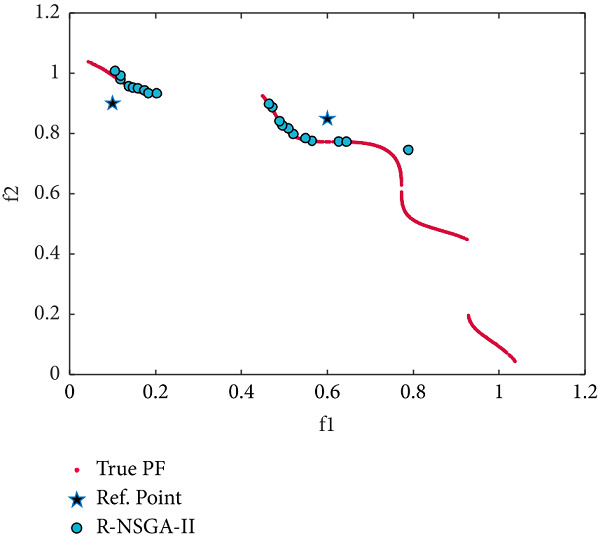
Efficient solutions obtained by R-NSGA-II on TNK with two RPs.

Finally, the desired regions obtained by the RS-KKTPM and R-NSGA-II on the TNK problem are illustrated in Figures 23 and 24, respectively. Two RPs and population members are chosen as displayed in Table 2. As shown in Figures 23 and 24, the performance of our approach is a little similar to R-NSGA-II. In summary, the introduced approach balances diversity and convergence around ROI for constraint test problems and handles any number of predefined RPs.

### 5.3. Experiments on Three-Objective Problems

**Figure 25:**
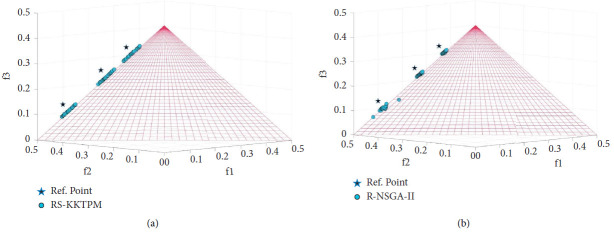
Comparison of performances on three-objective DTLZ1 with three RPs: (a) RS-KKTPM; (b) R-NSGA-II.

**Figure 26:**
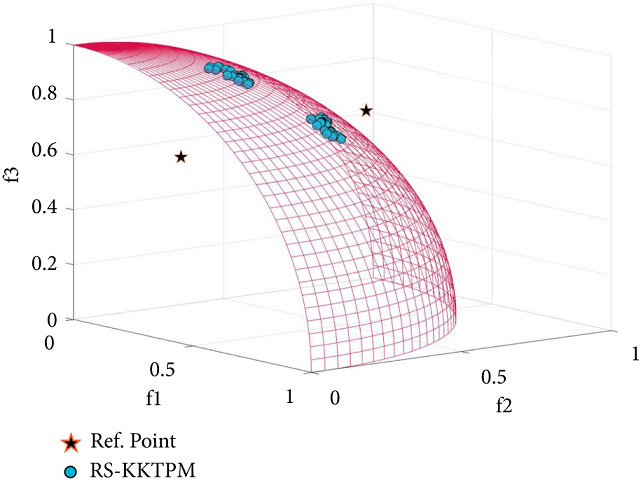
Efficient solutions obtained by RS-KKTPM on three-objective DTLZ2 with two RPs.

**Figure 27:**
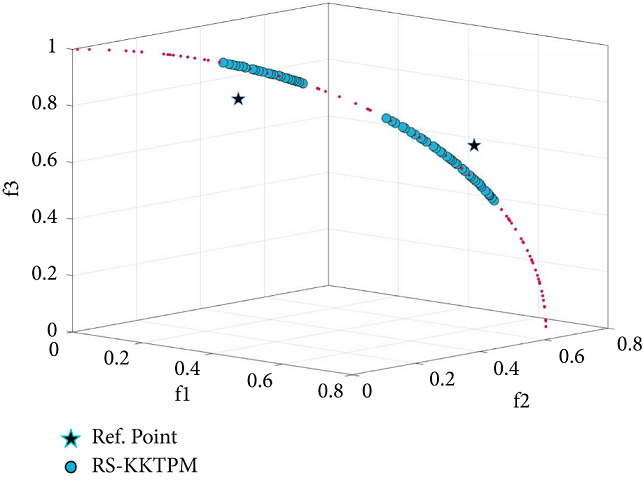
Efficient solutions obtained by RS-KKTPM on three-objective DTLZ5 with two RPs.

We will select the original DTLZ1, DTLZ2, and DTLZ5 and their scaled versions.  Table 3 provides some information about these problems and some parameters required. First, the DTLZ1 problem contains many local PFs, possibly causing some points to stop. This scenario is a relatively complicated problem to address for global optimality.  Figure 25 shows the obtained preferred PO solutions using RS-KKTPM and R-NSGA-II algorithms on the three-objective DTLZ1 (*DTLZ*1_3_) problem, and the parameter values are presented in Table 3. The three aspiration points are chosen. The distribution and convergence of solutions found by RS-KKTPM are substantially superior to those by R-NSGA-II, as displayed in Figure 25. Next, RS-KKTPM is utilized to solve the three-objective DTLZ2 problem.  Figure 26 shows the obtained solutions with 60 population members with two RPs.  Figure 26 clearly illustrates that the RS-KKTPM algorithm can access the efficient region of true PF with few number of population sizes, thereby helping the DM determine the required ROI easily. Finally, the RS-KKTPM is utilized to solve the three-objective DTLZ5 problem. The two RPs, *RP*_1_=(0.6,0.6,0.65)^*T*^ and *RP*_2_=(0.2,0.3,0.8)^*T*^, are used. The sizes of the preferred areas for *RP*_1_ and *RP*_2_ are 0.2 and 0.1, respectively, as shown in Table 3. Our algorithm is employed to solve this test problem with 60 populations and runs up to 300 generations. The obtained preferred areas of the true PO solutions are displayed in Figure 27. The obtained solutions are distributed according to the size of each preferred area. In a single simulation run, both areas are discovered. Note that the number of solutions generated in the first preferred area, corresponding to *RP*_1_, is greater than that generated in the second preferred area, corresponding to *RP*_2_. Well-convergent and well-distributed solutions are obtained on PF in all two RPs. Thus, the DM can control the size of each efficient region (s) of the true PF separately and in a single simulation run.

### 5.4. Experiments on Many-Objective Problems

**Figure 28:**
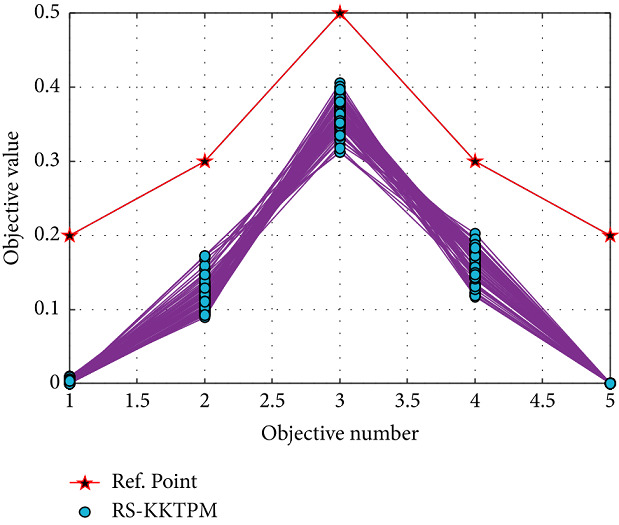
Efficient solutions obtained by RS-KKTPM on five-objective DTLZ1 with one RP.

**Figure 29:**
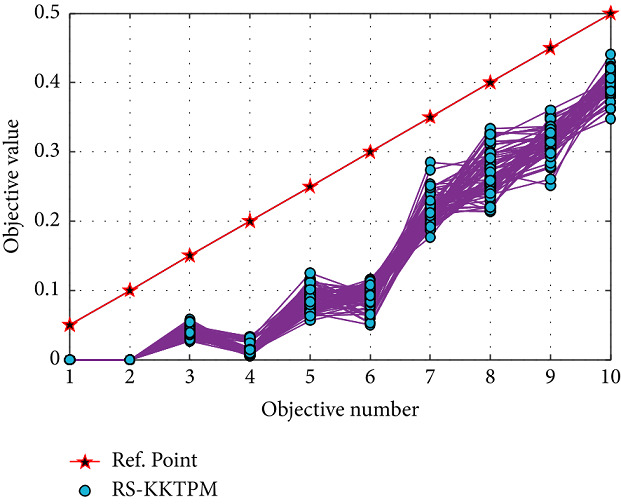
Efficient solutions obtained by RS-KKTPM on ten-objective DTLZ1 with one RP.

Finally, we test our introduced approach on the many-objective versions of the problems of DTLZ1 and DTLZ2.  Table 3 displays all parameters used for 5 and 10-objective problems. First, RS-KKTPM is used for *DTLZ*1_5_ and *DTLZ*1_10_ problems. Population sizes of 80 are used for the two problems. Figures 28 and 29 present the obtained part in a parallel coordinate plot. One RP is used for each problem, as shown in Table 3. The PO solutions of these problems must satisfy ∑_*i*=1_^*M*^*f*_*i*_=0.50. RS-KKTPM can discover the needful regions of the efficient set corresponding to the one predefined RP by the DM.

**Figure 30:**
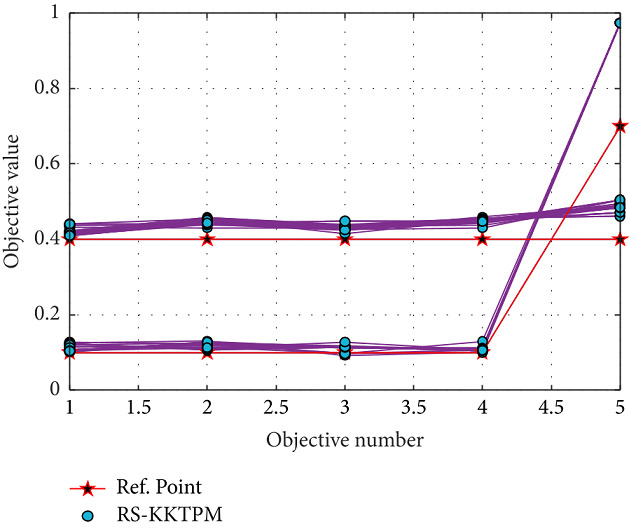
Efficient solutions obtained by RS-KKTPM on five-objective DTLZ2 with two RPs.

**Figure 31:**
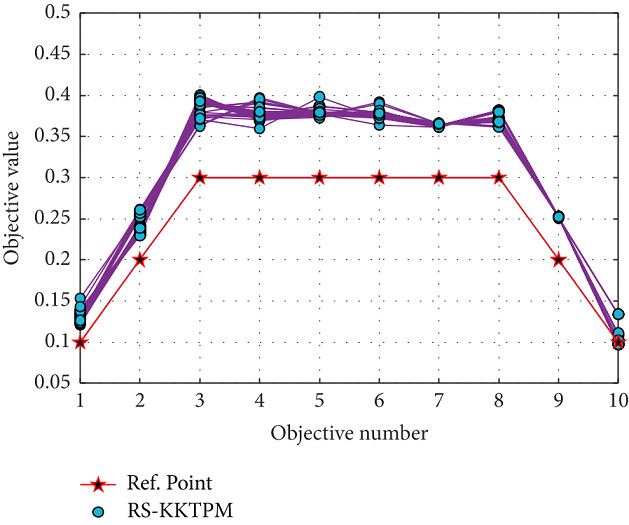
Efficient solutions obtained by RS-KKTPM on ten-objective DTLZ2 with one RP.

Finally, the RS-KKTPM algorithm is applied for *DTLZ*2_5_ and *DTLZ*2_10_ with 14 and 19 decision variables. This algorithm is applied for these test problems with 60 populations and runs up to 300 generations. Figures 30 and 31 show the obtained solutions for *DTLZ*2_5_ and *DTLZ*2_10_ with two and one aspiration points, respectively. The PO solutions to these problems must obey the next equation: ∑_*i*=1_^*M*^*f*_*i*_^2^=1. When computing the left side of this equation for all generated PO solutions, all the values lie in the range [1.000002, 1.000170] for *DTLZ*2_5_ problem and [1.000043, 1.002780] for *DTLZ*2_10_ problem, indicating that every solution is very close to the true PF. Thus, RS-KKTPM can converge to the PF corresponding to the chosen aspiration points.

### 5.5. Experiments on Engineering Problems

We now apply the RS-KKTPM to a couple of engineering design problems. The first test problem has two objectives, while the second test problem has three.

#### 5.5.1. Welded Beam Design Problem

**Figure 32:**
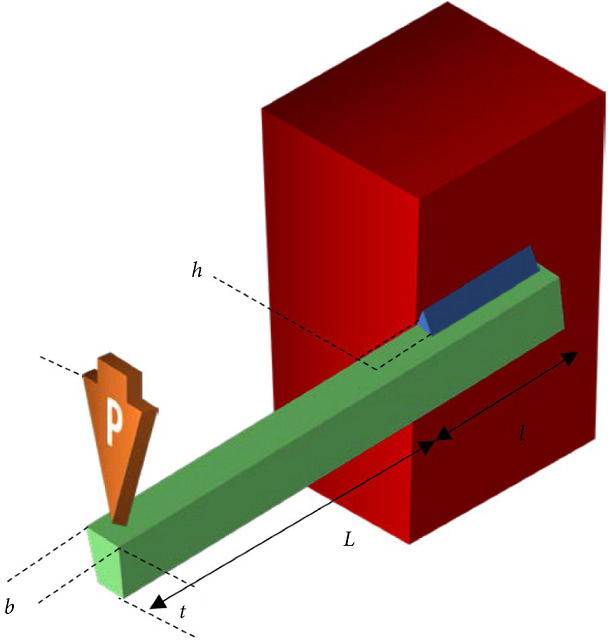
Welded beam design [52].

First, we now employ a two-objective welded beam design problem [48] as a real-world example. The first objective is to minimize the cost of fabrication, whereas the other objective is to minimize the end deflection of the welded beam. The design of welded beam structure is shown in Figure 32. This problem involves four decision variables, namely, *h* (weld thickness), *l* (clip length), *t* (the height of bar), and *b* (the thickness of bar). It has also four non-linear constraints. The problem is mathematically formulated as follows [48]:

(11) $$\begin{array}{c} minimize:\left\{\begin{array}{c} f_{1}\left(x\right)=1.10471h^{2}l+0.04811\left(\left(\frac{28}{2}\right)+l\right)tb\text{,}\\ f_{2}\left(x\right)=\frac{1372}{\left(625bt^{3}\right)}\text{,} \end{array}\right.\\ subject\;to\\ g_{1}\left(x\right)=\tau -13,600\leq 0,g_{2}\left(x\right)=\sigma -30,000\leq 0\text{,}\\ g_{3}\left(x\right)=h-b\leq 0,g_{4}\left(x\right)=6,000-P_{c}\leq 0\text{,}\\ 0.125\leq h,b\leq 5.0,and\;0.1\leq l,t\leq 10.0\text{,} \end{array}$$

where

(12) $$\begin{array}{c} \left\{\begin{array}{c} \upsilon =\frac{\left(6*10^{3}\right)}{\left(sqrt\left(2\right)*hl\right)}\text{,}\\ \kappa_{1}=\left(6*10^{3}\right)\left(\left(\frac{28}{2}\right)+\left(\frac{1}{2}\right)l\right)*\sqrt{\left(\left(\frac{1}{4}\right)\left(l^{2}+{\left(t+h\right)}^{2}\right)\right)}\text{,}\\ \kappa_{2}=\left(\frac{707}{1000}\right)hl\left(\frac{l^{2}}{12}+\left(\frac{1}{4}\right)\left(l^{2}+{\left(t+h\right)}^{2}\right)\right)\text{,}\\ \alpha =\frac{\kappa_{1}}{2\kappa_{2}}\text{,}\\ \beta =\frac{l\;\upsilon \alpha }{sqrt\left(\left(1/4\right)\left(l^{2}+{\left(t+h\right)}^{2}\right)\right)}\\ \tau =\sqrt{\left(\upsilon^{2}+\alpha^{2}+\beta \right)}\text{,}\\ \sigma =\frac{\left(504*10^{3}\right)}{\left(bt^{2}\right)}\text{,}\\ P_{c}=\left(\frac{43*514243}{36}\right)*sqrt\sqrt{\left(\frac{b^{6}*t^{2}}{\left(144/4\right)}\right)}\left(1.0-t\left(\frac{2680}{94919}\right)\right)\text{.} \end{array}\right. \end{array}$$

**Figure 33:**
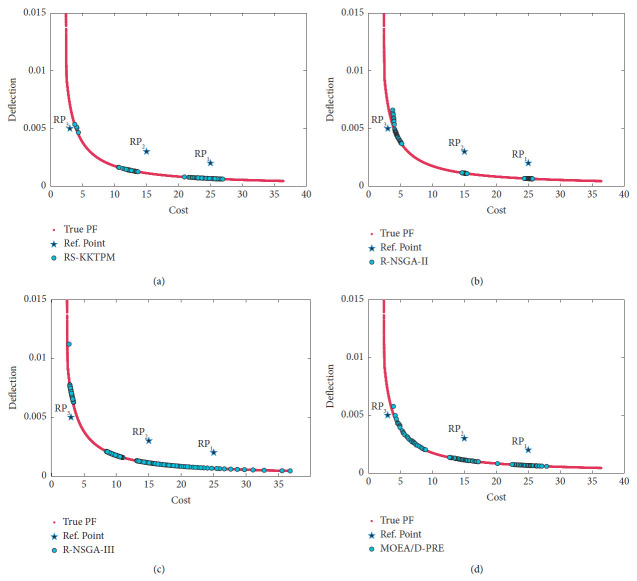
Comparison of performances on the welded beam design problem with three RPs: (a) RS-KKTPM; (b) R-NSGA-II; (c) R-NSGA-III; (d) MOEA/D-PRE.

Figure 33 shows the efficient solutions produced by RS-KKTPM, R-NSGA-II, R-NSGA-III, and MOEA/D-PRE [30], respectively, to the welded beam design problem. The relevant parameters of the four comparison algorithms are briefly presented below. Three RPs are chosen: *RP*_1_ = (3, 0.005), *RP*_2_ = (15, 0.003), and *RP*_3_ = (25, 0.002). The comparison algorithms are used with 100 population members and run for 200 generations. For RS-KKTPM approach, the radius corresponding to each RP is *δ*_1_ = 0.2, *δ*_2_ = 0.1, and *δ*_3_ = 0.05. For the rest of the algorithms, the size of preferred regions is equal to 0.1. Note that the proposed algorithm can control the size of each region separately, while other algorithms cannot. In this problem, all objective function values are normalized using the ideal point as the lower bound and the nadir point estimation as the upper bound to stay within the interval [0, 1]. We used (0, 0) as the ideal point and (36, 0.015) as the nadir point.

Figure 33(a) shows that the introduced algorithm outperforms the others by adjusting the size of each preferred region (separately) corresponding to the supplied RP. It has the ability to steer the solutions towards the preferred regions in proportion to the size of each area. As shown in Figure 33(a), the obtained solutions in the preferred region, corresponding to *RP*_1_, are more compared to the obtained solutions in the other two preferred regions. Indeed, the size of the ROI, corresponding to *RP*_1_, is greater than the sizes of the other two preferred regions, i.e., *δ*_1_ > *δ*_2_ and *δ*_1_ > *δ*_3_. On the other hand, the preferred region, corresponding to *RP*_3_, contains a few solutions compared to the other two preferred regions because *δ*_3_ < *δ*_1_> and *δ*_3_ > *δ*_2_. Figure 33(a) also shows that the RS-KKTPM can produce well-distributed solutions along the preferred part. The advantages of the RS-KKTPM, discussed above, are mostly not found in R-NSGA-II, R-NSGA-III, and MOEAD-PRE (see Figures 33(b)–33(d)). In summary, if the DM is interested in finding PO solutions in three main areas (intermediate cost and deflection, minimum cost, and minimum deflection), the introduced algorithm can find solutions near the given RPs, rather than finding solutions on the whole PF, allowing the DM to deal with only a few solutions that lie in parts of her/his interest. Moreover, if the DM is interested in finding these solutions within different sizes for all regions, the proposed algorithm can provide them.

#### 5.5.2. Car Side Impact Design Problem

**Figure 34:**
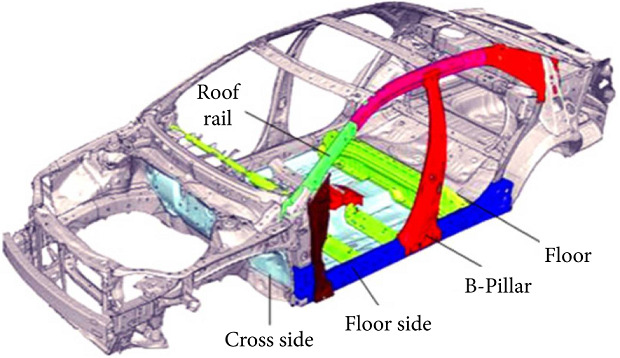
Car side impact design [53].

Car side impact design is a constrained optimization problem [49]. This problem has three optimization objectives which are described as follows: the first is to reduce the car's weight, the second objective is to minimize the pubic force experienced by a passenger, and the last objective is to minimize the average velocity of the V-Pillar responsible for withstanding the impact load. It has seven decision variables: B-Pillar, door beam, B-Pillar inner reinforcement, floor side inner, door beltline reinforcement, cross members, and roof rail (see Figure 34). The mathematical model of this problem is as follows [49]:

(13) $$\begin{array}{c} minimize\text{:}\\ \left\{\begin{array}{c} f_{1}\left(x\right)=1.98+4.9v_{1}+6.67v_{2}+6.98v_{3}+4.01v_{4}+1.78v_{5}+0.00001v_{6}+2.73v_{7},\\ f_{2}\left(x\right)=4.72-0.5v_{4}-0.19v_{2}v_{3},\\ f_{3}\left(x\right)=0.5\left(10.58-0.674v_{1}v_{2}-0.67275v_{2}+16.45-0.489v_{3}v_{7}-0.843v_{5}v_{6}\right), \end{array}\right.\\ subject\;to\\ g_{1}\left(x\right)=1.160-0.3717v_{2}v_{4}-0.0092928v_{3}\leq 1.0\text{,}\\ g_{2}\left(x\right)=0.261-0.0159v_{1}v_{2}-0.06486v_{1}-0.019v_{2}v_{7}\\ +0.0144v_{3}v_{5}+0.0154464v_{6}\leq 0.32\text{,}\\ g_{3}\left(x\right)=0.214+0.00817v_{5}-0.045195v_{1}-0.0135168v_{1}\\ +0.03099v_{2}v_{6}-0.018v_{2}v_{7}+0.007176v_{3}\\ +0.023232v_{3}-0.00364v_{5}v_{6}-0.018v_{2}v_{2}\leq 0.32\text{,}\\ g_{4}\left(x\right)=0.740-0.610v_{2}-0.031296v_{3}-0.031872v_{7}+0.227v_{2}v_{2}\leq 0.32\text{,}\\ g_{5}\left(x\right)=8.980+3.8180v_{3}-4.2v_{1}v_{2}+1.27296v_{6}-2.68065v_{7}\leq 0.32\text{,}\\ g_{6}\left(x\right)=33.860+2.95v_{3}-5.057v_{1}v_{2}-3.795v_{2}-3.4431v_{7}\\ +1.45728\leq 0.32,\\ g_{7}\left(x\right)=46.360-9.90v_{2}-4.4505v_{1}\leq 0.32\text{,}\\ g_{8}\left(x\right)=4.720-0.50v_{4}-0.190v_{2}v_{3}\leq 4.0\text{,}\\ g_{9}\left(x\right)=10.58-0.674v_{1}v_{2}-0.67275v_{2}\leq 9.9\text{,}\\ g_{10}\left(x\right)=16.45-0.489v_{3}v_{7}-0.843v_{5}v_{6}\leq 15.7\text{,}\\ 0.5\leq v_{1,3,4}\leq 1.5,and\;0.45\leq v_{2}\leq 1.35,0.875\leq v_{5}\leq 2.625,and\;0.4\leq v_{6,7}\leq 1.2\text{.} \end{array}$$

**Figure 35:**
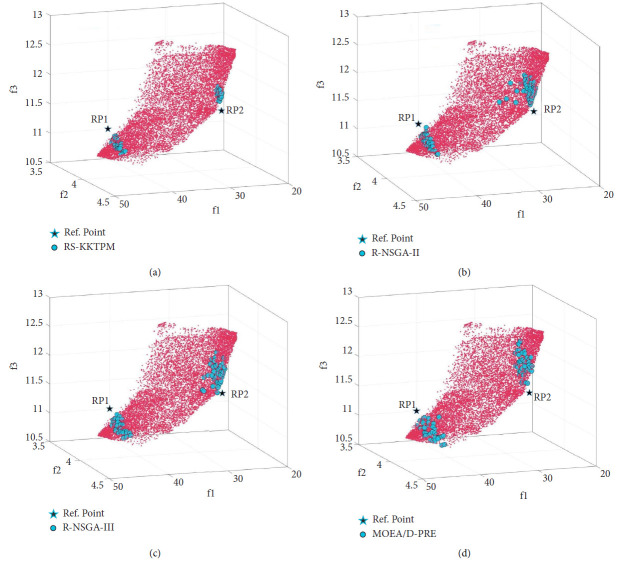
Comparison of performances on the car side impact problem with two RPs: (a) RS-KKTPM; (b) R-NSGA-II; (c) R-NSGA-III; (d) MOEA/D-PRE.

Figure 35 shows the solutions produced by RS-KKTPM, R-NSGA-II, R-NSGA-III, and MOEAD-PRE on the car side impact design problem. In this problem, all relevant parameters of the four comparison approaches are briefly presented below. Two RPs are chosen: RP1 = (40, 3.5, 11) and RP2 = (26, 4, 11.5). For this test problem, the comparison algorithms are used with 80 population members and run until 300 generations. For the RS-KKTPM algorithm, the radius corresponding to each RP is *δ*_1_ = 0.1 and *δ*_2_ = 0.05. For the rest of the algorithms, the size of preferred regions is equal to 0.1. In this problem, all objective function values are normalized using the ideal point (15, 3, 10) and nadir point (50, 5, 14).  Figure 35(a) shows that the suggested algorithm can control the number of solutions for each desired area in proportion to its size. In other words, a larger desired area gets more solutions, while a smaller preferred region gets fewer solutions. As displayed in Figure 35(a), the number of solutions for the first preferred area, corresponding to the *P*_1_, is more than that for the second desired area, corresponding to the *RP*_2_. This is because the size of the first region is greater than the size of the second region, i.e., *δ*_1_ > *δ*_2_. Therefore, RS-KKTPM can control the size of each desired area separately, while the rest of its algorithms cannot do that (see Figures 35(b)–35(d)). Thus, if the DM is interested in finding solutions in region of different sizes, the introduced algorithm can find solutions near the RPs and proportional to the size of each preferred area separately.

### 5.6. Performance Metrics

No single performance metric can provide an accurate assessment of an EMO's performance [54]. In our empirical investigations, we use two of the most recognized performance metrics to assess the quality of preferable efficient solutions of preference-based EMO algorithms: R-HV and R-IGD. [55]. Both metrics are utilized to detect the ROI's convergence and the diversity of efficient solutions simultaneously. They are based on two performance metrics, the hypervolume (HV) metric and the inverted generational distance (IGD) metric, which are designed for the entire PF and applicable for partial preferable efficient solutions. The larger the R-HV values are or the smallest the R-IGD values are, the better the performance of the tested algorithm is. Additional details can be found in Li et al. [55].

### 5.7. Performance Comparison with Other Preference-Based EMO Algorithms

We compare RS-KKTPM with six EMO preference algorithms, including R-NSGA-II, g-NSGA-II, r-NSGAII, R-NSGA-III, WV-MOEA-P, and MOEA/D-PRE, to verify RS-KKTPM performance. We determine the parameters of the mentioned algorithms in advance to approximate a similar ROI and make the experimental findings comparable. The parameters utilized in the comparative study are summarized as follows:

- Reproduction operators: in all simulations, crossover probability = 0.9, mutation probability = 1/*n*, distribution index for SBX operator = 10, and distribution index for polynomial mutation operator = 20.
- Number of evaluations, population size, and RP coordinate setting: different parameters for a set of different test instances are displayed in Table 4.
- Number of runs: it is 21 for all algorithms on all test problems.
- Size of the preferred region: it is 0.1 for all algorithms on all test problems.
- Parameters in r-NSGAII: the weight vector *w* was set as (1/*M*, 1/*M*,…).

As mentioned earlier, S-KKTPM requires the gradient of all objective and constraint functions. Then, algebraic calculations are performed to compute the theoretical closeness of **x** to the true optimal solution. For MOPs, S-KKTPM calculates the closeness metric from a specific PO point. In this article, the introduced RS-KKTPM approach is then compared with six EMO approaches. For a fair comparison between all algorithms, we used equal function evaluations in the comparison.

Compared to the number of function evaluations required for a solution evaluation, the savings reported for the S-KKTPM calculation may not be significant because the evaluation of S-KKTPM for all solutions is an additional computational expense and requires more computation. Thus, once gradients are computed for a real-world problem, the computational time needed for the S-KKTPM optimization procedure would make a small addition to the overall computational time. In the meantime, S-KKTPM helps improve convergence and can differentiate between different non-dominated solutions that are not applicable by using the Euclidean distance or any other evolutionary algorithm.

Tables 5 and 6 display the mean and standard deviation of R-HV and R-IGD values, respectively. The best mean of R-HV and R-IGD metrics is highlighted in bold in Tables 5 and 6.

According to R-HV metric, the ROI is approximated by the introduced RS-KKTPM algorithm in a better way than other algorithms for all the examined problems except the ZDT4, *DTLZ*2_3_, and *DTLZ*2_5_ test cases (see Table 5). We obtain almost the same results according to the R-IGD metric, as shown in Table 6. Based on the R-HV values and the R-IGD values, RS-KKTPM demonstrates better distribution and convergence than other algorithms. The practical findings on the 14 benchmark test problems illustrate that the RS-KKTPM approach outperforms the other approaches used in 11 of 14 comparisons.

## 6. Conclusions

In this study, the RS-KKTPM preference-based EMO algorithm is proposed. It is an expansion of the R-NSGA-II method, where the Euclidean distance metric is replaced by the S-KKTPM metric. The following are the properties of this new algorithm:

- The RS-KKTPM can obtain the ROI at any specific position of the RP (in the feasible area, on/near the PF, and the infeasible area).
- The range of each obtained ROI can be controlled by adjusting the interest radius size of each ROI separately and in a single simulation run.
- The RS-KKTPM algorithm, given herein, improves the quality of the PF approximation and allows a uniform distribution of the approximating objective vectors.
- The performance of RS-KKTPM is better than that of R-NSGA-II, g-NSGA-II, r-NSGAI, R-NSGA-III, WV-MOEA-P, and MOEA/D-PRE on most multi and many-objective problems.

The direction of future research focuses on using the S-KKTPM metric to improve the performance of other EMO optimization algorithms by reference direction approaches, such as MOEA/D and NSGA-III. These approaches can also be utilized to solve engineering design problems and highly complex problems.

## Figures and Tables

**Algorithm 1:**
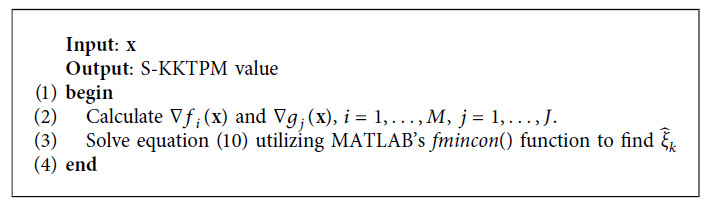
S-KKTPM pseudocode.

**Algorithm 2:**
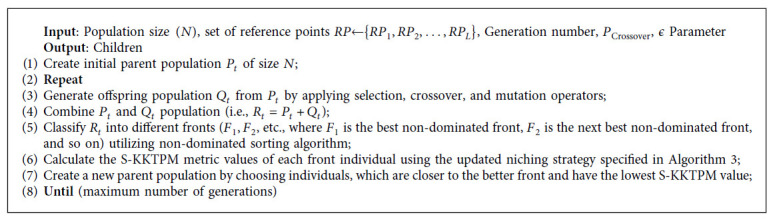
RS-KKTPM pseudocode.

**Algorithm 3:**
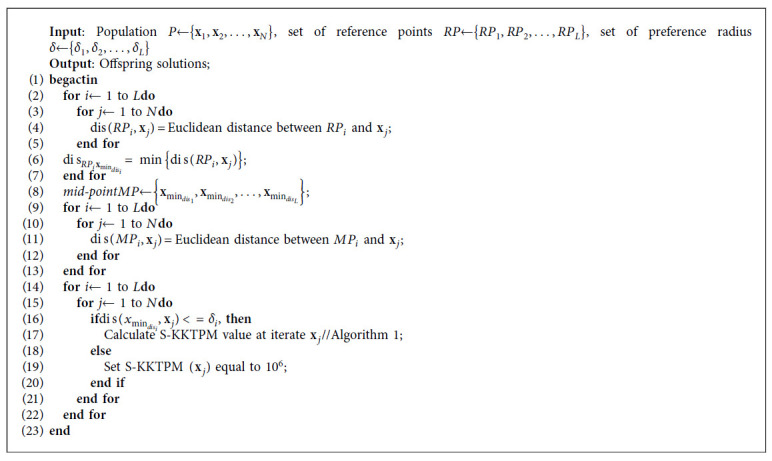
RS-KKTPM niching strategy.

**Table 1:** List of nomenclature and abbreviations.

Nomenclature	
*n*	Number of decision variables
*M*	Number of objective functions
*J*	Number of inequality constraints
*P*	Number of equality constraints
≺	Pareto-dominance relation
Ω	Feasible search space
*δ*	Radius of the ROI
*ℝ* ^*M*^	Objective space
*N*	Population size
*ℝ* ^*n*^	Decision variable space
*β*_*i*_	Lagrange multiplier of *i*th objective function
*γ*_*j*_	Lagrange multiplier if *j*th inequality constraint
*ξ*_*k*_	KKTPM value
$\overset{\wedge }{\xi_{k}}$	S-KKTPM value
*P*_*c*_	Crossover probability
*P*_*m*_	Mutation probability
*ϵ*	Size of the preferred region

*Abbreviations*	
BNH	Constrained bi-objective test problem by Binh and Korn
DM	Decision maker
DTLZ	Deb–Thiele–Laumanns–Zitzler test problem set
EMO	Evolutionary multi-objective optimization
g-NSGA-II	g-Dominance relation-based non-dominated sorting genetic algorithm-II
KKT	Karush–Kuhn–Tucker
KKTPM	KKT proximity metric
S-KKTPM	Simplified KKT proximity metric
MCDM	Multi-criteria decision making
MOP	Multi-objective optimization problem
MOEA/D	Multi-objective evolutionary algorithm based on decomposition
MOEA/D-PRE	MOEA/D based on preference information
NSGA-II	Non-dominated sorting genetic algorithm-II
NSGA-III	Non-dominated sorting genetic algorithm-III
OSY	Constrained bi-objective test problem by Osyczka and Kundu
PF	Pareto front
PO	Pareto-optimal
PS	Pareto set
R-IGD	R-metric based on inverse generational distance
R-HV	R-metric based on hypervolume
r-NSGA-II	r-Dominance relation-based NSGA-II
R-NSGA-II	Reference point-based NSGA-II
R-NSGA-III	Reference point-based NSGA-III
ROI	Region of interest
RP	Reference point
RS-KKTPM	Reference point based on S-KKTPM
SBX	Simulated binary crossover
SRN	A constrained bi-objective test problem by Srinivas and Deb
SPEA2	Improved version of strength Pareto evolutionary algorithm
TNK	A constrained bi-objective test problem by Tanaka
WV-MOEA-P	Weight vector-based multi-objective optimization algorithm with preference
ZDT	Zitzler–Deb–Thiele test problem set

**Table 2:** Parameters: columns from left to right represent problem name, number of objectives, number of variables, population size, maximum number of generations, reference points, and size of region of interest.

Problem	nobj	nvar	Popsize	MaxGen	Ref. point	Size of ROI
	**M**	**n**	**N**		$\overset{⟶}{RP}$	*δ*
ZDT1	2	30	40	200	*RP*_1_ = (0.0, 0.8)	0.05
					*RP*_2_ = (0.2, 0.6)	0.05
					*RP*_3_ = (0.3, 0.3)	0.05
					*RP*_4_ = (0.7, 0.25)	0.05
					*RP*_5_ = (0.9, 0.0)	0.05

ZDT2	2	30	40	200	*RP*_1_ = (0.9, 0.5)	0.1
					*RP*_2_ = (0.3, 0.8)	0.1

ZDT3	2	30	40	200	*RP*_1_ = (0.1, 0.4)	0.1
					*RP*_2_ = (0.5, 0.4)	0.1
					*RP*_3_ = (0.7, −0.25)	0.1

ZDT4	2	10	80	500	*RP*_1_ = (0.6, 0.6)	0.15

ZDT6	2	10	40	200	*RP*_1_ = (0.3, 0.8)	0.03
					*RP*_2_ = (0.9, 0.4)	0.05
					*RP*_3_ = (0.64, 0.59)	0.1

BNH	2	2	40	200	*RP*_1_ = (10, 20)	0.1
					*RP*_2_ = (60, 15)	0.1
					*RP*_3_ = (90, 5)	0.1

SRN	2	2	40	200	*RP*_1_ = (170, −200)	0.1
					*RP*_2_ = (100, −50)	0.1

OSY	2	6	40	200	*RP*_1_ = (−230, 40)	0.05
					*RP*_2_ = (−100, 15)	0.1

TNK	2	2	20	200	*RP*_1_ = (0.1, 0.90)	0.1
					*RP*_2_ = (0.6, 0.85)	0.1

**Table 3:** Parameters: columns from left to right represent the problem name, number of objectives, number of variables, population size, maximum number of generations, reference points, and size of region of interest.

Problem	nobj	nvar	Popsize	MaxGen	Ref. point	Size of ROI
	**M**	**n**	**N**		$\overset{⟶}{RP}$	*δ*
DTLZ1	3	5	80	300	*RP*_1_ = (0.0, 0.25, 0.3)	0.05
					*RP*_2_ = (0.0, 0.4, 0.15)	0.05
					*RP*_3_ = (0.0, 0.15, 0.4)	0.05
	5	7	80	300	*RP*_1_ = (0.2, 0.3, 0.5, 0.3, 0.2)	0.05
	10	12	80	300	*RP*_1_ = (0.05, 0.1, 0.15, 0.20, 0.25, 0.30, 0.35, 0.4, 0.45, 0.5)	0.1

DTLZ2	3	8	60	300	*RP*_1_ = (0.6, 0.5, 0.8)	0.05
					*RP*_2_ = (0.2, 0.2, 0.6)	0.05
	5	14	60	300	*RP*_1_ = (0.4, 0.4, 0.4, 0.4, 0.4)	0.02
					*RP*_2_ = (0.1, 0.1,0.1, 0.1, 0.7)	0.02
	10	19	60	300	*RP*_1_ = (0.1, 0.2, 0.3, 0.3, 0.3, 0.3, 0.3, 0.3, 0.2, 0.1)	0.05

DTLZ5	3	12	60	300	*RP*_1_ = (0.6, 0.6, 0.65)	0.2
					*RP*_2_ = (0.2, 0.3, 0.8)	0.1

**Table 4:** Parameter setting of the test instances utilized in preference-based EMO algorithms.

Problem	Ref. point	Popsize	Function evaluations
*ZDT*1	(0.4, 0.5)	40	8000
*ZDT*2	(0.9, 0.4)	40	8000
*ZDT*3	(0.1, 0.4)	40	8000
*ZDT*4	(0.6, 0.6)	80	40000
*ZDT*6	(0.5, 0.7)	40	8000
*DTLZ*1_3_	(0.0, 0.4, 0.15)	80	24000
*DTLZ*2_3_	(0.6, 0.5, 0.8)	60	18000
*DTLZ*5_3_	(0.2, 0.3, 0.8)	60	18000
*DTLZ*1_5_	(0.2, 0.3, 0.5, 0.3, 0.2)	80	24000
*DTLZ*2_5_	(0.4, 0.4, 0.4, 0.4, 0.4)	60	18000
*DTLZ*1_10_	(0.05, 0.10, 0.15, 0.20, 0.25, 0.30, 0.35, 0.4, 0.45, 0.5)	80	24000
*DTLZ*2_10_	(0.1, 0.2, 0.3, 0.3, 0.3, 0.3, 0.3, 0.3, 0.2, 0.1)	60	18000
Welded	(15, 0.0030)	100	20000
CAR	(40, 4, 11)	80	24000

**Table 5:** Findings of mean and standard deviation for R-HV values utilizing several preference-based EMO algorithms.

Problem	RS-KKTPM	R-NSGA-II	g-NSGA-II	r-NSGA-II	R-NSGA-III	WV-MOEA-P	MOEA/D-PRE
*ZDT*1	**4.5121 (4.35*e − *2)**	4.4507 (8.75*e − *2)	4.2776 (1.88*e − *1)	4.2303 (9.67*e − *2)	4.3990 (2.66*e − *2)	2.1604 (4.50*e − *1)	4.1497 (2.44*e − *1)
*ZDT*2	**4.5090 (5.57*e − *2)**	4.4241 (1.32*e − *1)	4.4231 (1.58*e − *1)	4.2033 (1.08*e − *1)	3.8722 (2.83*e − *1)	1.8046 (9.27*e − *2)	4.1300 (1.96*e − *1)
*ZDT*3	**3.7582 (2.88*e − *2)**	3.7009 (2.06*e − *2)	3.5993 (1.2*e − *1)	3.2484 (2.52*e − *1)	3.0962 (2.81*e − *1)	2.5306 (4.11*e − *1)	3.4078 (3.56*e − *1)
*ZDT*4	5.0791 (1.71*e − *1)	5.0525 (1.69*e − *1)	**5.2728 (9.86*e − *3)**	4.0871 (2.77*e − *2)	5.0714 (8.40*e − *3)	1.5734 (6.56*e − *1)	5.1849 (2.40*e − *2)
*ZDT*6	**4.0303 (2.57*e − *2)**	3.9785 (5.70*e − *2)	3.8749 (1.12*e − *1)	3.9565 (1.17*e − *1)	2.7656 (5.55*e − *1)	1.4462 (1.25*e + *0)	3.9088 (5.65*e − *1)
*DTLZ*1_3_	**8.3033 (1.10*e − *2)**	8.0683 (6.85*e − *2)	5.4812 (0.00*e + *0)	8.1306 (8.30*e − *1)	8.1918 (4.65*e − *3)	7.8932 (6.79*e − *3)	8.2689 (1.31*e − *1)
*DTLZ*2_3_	9.2300 (1.59*e − *1)	9.0270 (5.33*e − *2)	9.1524 (7.71*e − *2)	9.1859 (1.18*e − *1)	9.6399 (6.57*e − *4)	9.1626 (8.06*e − *3)	**9.8589 (7.14*e − *2)**
*DTLZ*5_3_	**7.4794 (3.86*e − *2)**	7.1209 (9.84*e − *2)	7.0461 (6.16*e − *2)	6.9673 (1.12*e − *1)	7.3971 (3.59*e − *2)	7.3473 (1.00*e − *2)	7.4601 (4.93*e − *1)
*DTLZ*1_5_	**51.8760 (1.50*e + *0)**	50.5049 (1.38*e + *0)	—	31.9832 (14.14*e + *0)	46.7584 (1.06*e − *1)	44.9438 (6.13*e − *2)	47.9684 (6.82*e − *1)
*DTLZ*2_5_	29.4583 (1.10*e + *0)	28.6195 (5.47*e − *2)	4.3340 (3.19*e + *0)	29.2152 (7.47*e − *1)	**31.0704 (1.37*e + *0)**	29.2568 (1.41*e − *1)	30.5327 (1.41*e + *0)
*DTLZ*1_10_	**1486.5990 (109.92*e + *0)**	1412.7149 (119.32*e + *0)	0.0 (0.0)	6.6096 (11.112*e + *0)	1342.6744 (8.24*e + *0)	1260.3203 (11.03*e + *0)	1303.7348 (25.98*e + *0)
*DTLZ*2_10_	**729.1487 (40.01*e + *0)**	704.7066 (2.57*e + *0)	571.4168 (116.75*e + *0)	7.6764 (12.83*e + *0)	664.9139 (5.08*e + *0)	594.3837 (24.46*e + *0)	445.8092 (93.27*e + *0)
*Welded*	**14.0481 (3.769*e − *01)**	13.9529 (1.94*e − *3)	4.0074 (8.37*e − *05)	4.0077 (5.07*e − *08)	13.9654 (6.05*e − *4)	4.1616 (5.72*e − *3)	9.1082 (11.32*e + *0)
*CAR*	**10.0671 (8.720*e − *01)**	8.2522 (8.28*e − *1)	1.0275 (5.07*e + *0)	2.2254 (1.52*e + *0)	9.9254 (5.05*e − *2)	6.5282 (5.96*e + *0)	8.7214 (4.72*e − *1)

**Table 6:** Findings of mean and standard deviation for R-IGD values utilizing several preference-based EMO algorithms.

Problem	RS-KKTPM	R-NSGA-II	g-NSGA-II	r-NSGA-II	R-NSGA-III	WV-MOEA-P	MOEA/D-PRE
*ZDT*1	**0.0152 (6.88*e − *3)**	0.0275 (1.05*e − *2)	0.0677 (3.64*e − *2)	0.0705 (2.05*e − *2)	0.0339 (6.90*e − *3)	0.88289 (2.40*e − *1)	0.0990 (5.68*e − *2)
*ZDT*2	**0.0159 (1.11*e − *2)**	0.0311 (3.55*e − *2)	0.0431 (2.91*e − *2)	0.0776 (1.76*e − *2)	0.1770 (8.46*e − *2)	1.0425 (4.92*e − *2)	0.1082 (5.22*e − *2)
*ZDT*3	**0.0155 (8.17*e − *3)**	0.0198 (7.79*e − *3)	0.0357 (3.01*e − *2)	0.1510 (9.05*e − *2)	0.2085 (1.22*e − *1)	0.4436 (1.85*e − *1)	0.1103 (1.28*e − *2)
*ZDT*4	0.0366 (3.62*e − *2)	0.0409 (3.72*e − *2)	**0.0056 (1.83*e − *3)**	0.2972 (6.56*e − *3)	0.0236 (1.73*e − *3)	1.1441 (4.00*e − *1)	0.0095 (1.95*e − *3)
*ZDT*6	**0.0305 (6.01*e − *3)**	0.0468 (1.10*e − *2)	0.0728 (3.27*e − *2)	0.0555 (2.43*e − *2)	0.4645 (2.22*e − *1)	1.4101 (9.86*e − *1)	0.0986 (1.80*e − *1)
*DTLZ*1_3_	**0.0805 (4.54*e − *3)**	0.1003 (5.68*e − *3)	0.4525 (0.00*e + *0)	0.0959 (9.95*e − *2)	0.0814 (1.40*e − *3)	0.1091 (8.56*e − *3)	0.0830 (1.56*e − *2)
*DTLZ*2_3_	0.0598 (9.62*e − *3)	0.0673 (3.50*e − *3)	0.0318 (6.197*e − *3)	0.0597 (1.04*e − *2)	**0.0210 (6.89*e − *5)**	0.0532 (3.19*e − *4)	0.0313 (5.05*e − *3)
*DTLZ*5_3_	**0.0834 (1.39*e − *3)**	0.1011 (5.32*e − *3)	0.1254 (4.12*e − *3)	0.1144 (8.83*e − *3)	0.0865 (1.80*e − *3)	0.0903 (7.70*e − *4)	0.0857 (3.97*e − *2)
*DTLZ*1_5_	**0.1125 (1.61*e − *2)**	0.1507 (1.91*e − *2)	—	0.6338 (6.84*e − *1)	0.1425 (1.02*e − *3)	0.18415 (1.20*e − *3)	0.16923 (8.06*e − *3)
*DTLZ*2_5_	0.1388 (1.79*e − *2)	0.1536 (7.06*e − *4)	1.4854 (3.70*e − *1)	0.1408 (8.11*e − *2)	**0.0930 (2.94*e − *2)**	0.12086 (3.50*e − *3)	0.1453 (1.86*e − *2)
*DTLZ*1_10_	**0.7036 (4.60*e − *2)**	0.7324 (6.14*e − *2)	447.2251 (222.61*e + *0)	4.6381 (1.78*e + *0)	0.7203 (9.92*e − *3)	0.75856 (4.06*e − *3)	0.7697 (1.23*e − *2)
*DTLZ*2_10_	**0.2621 (1.52*e − *2)**	0.3456 (2.76*e − *3)	1.0850 (1.26*e − *1)	3.6118 (8.26*e − *1)	0.30169 (1.30*e − *2)	0.4210 (5.62*e − *2)	0.4701 (7.94*e − *2)
*Welded*	**0.9001 (2.62*e − *1)**	0.9402 (5.63*e − *1)	4.400 (1.02*e + *0)	3.2354 (1.26*e + *0)	0.9358 (3.02*e − *5)	1.912 (2.60*e + *0)	1.0105 (3.94*e − *1)
*CAR*	**1.5211 (5.21*e − *1)**	1.9402 (7.53*e − *1)	5.2504 (22.02*e + *0)	3.2356 (26.28*e + *0)	1.5564 (6.02*e − *1)	2.0502 (2.92*e − *1)	1.8502 (5.65*e − *1)

## Data Availability

No data were used to support this study.
